# Physiochemical and functional evaluation of the first-in-class anti-cancer IgE antibody drug, MOv18, through process development and good manufacturing practice production

**DOI:** 10.1080/19420862.2025.2451295

**Published:** 2025-01-20

**Authors:** Heather J Bax, Jitesh Chauhan, Alexandra J McCraw, Melanie Grandits, Chara Stavraka, Heike Lentfer, Tim Hillyer, Simon Carroll, Kim Vigor, Chris Selkirk, Mariangela Figini, Jack Cheeseman, Paulina A Urbanowicz, Richard A Gardner, Daniel I R Spencer, Nigel Westwood, Sarah Mellor, James Spicer, Debra H Josephs, Sophia N Karagiannis

**Affiliations:** a St. John’s Institute of Dermatology, School of Basic & Medical Biosciences & KHP Centre for Translational Medicine, Guy’s Hospital, King’s College London, London, UK; b Centre for Drug Development, Cancer Research UK, London, UK; c ANP2, Department of Advanced Diagnostics, Fondazione IRCCS, Istituto Nazionale dei Tumori, Milan, Italy; d Ludger, Ltd., Culham Campus, Abingdon, Oxfordshire, UK; e School of Cancer and Pharmaceutical Sciences, King’s College London, London, UK; f Cancer Centre, Guy’s and St Thomas’ NHS Foundation Trust, London, UK; g Breast Cancer Now Research Unit, School of Cancer & Pharmaceutical Sciences, King’s College London, Innovation Hub, Guy’s Cancer Centre, London, UK

**Keywords:** AllergoOncology, antibody immunotherapy, basophil activation test, cancer, good manufacturing practice, IgE, investigational medicinal product, MOv18 IgE, process development, production

## Abstract

Antibodies used for cancer therapy are monoclonal IgGs, but tumor-targeting IgE antibodies have shown enhanced effector cell potency against cancer in preclinical models. Research-grade recombinant IgE antibodies have been generated and studied for several decades. The recent Phase 1 clinical trial of the first-in-class MOv18 IgE, however, necessitated the inaugural process development and scaled manufacture of a recombinant IgE to clinical quality standards. During the process development and scaled Good Manufacturing Practice production, we demonstrate the retention of glycosylation state, biophysical profile, and functional characteristics of MOv18 IgE, including Fc-mediated mast cell degranulation and tumor cell killing. Assessment of manufacturing parameters shows expected pH, purity, concentration, and stability properties, as well as below threshold levels of known biological manufacturing contaminants. We confirm the suitability of the pipeline described for generating intact, functionally active, clinical-grade material for this novel therapeutic class as an Investigational Medicinal Product (IMP), with comparable characteristics to the original research-grade antibody. Furthermore, we screened patient blood *ex vivo* for potential type I hypersensitivity reaction to MOv18 IgE, using the basophil activation test, to identify patients not predicted to be hypersensitive to MOv18 IgE administration. This study supports the production of functionally active clinical grade (IMP) recombinant IgE and paves the way for the development of a new therapeutic antibody class for a range of antigenic specificities and disease settings.

## Introduction

Although clinically used tumor-targeting IgG monoclonal antibodies can be efficacious cancer therapeutics for many cancer types, their effectiveness in engaging the immune system against tumors may be restricted in some patients.^1^ The recent development of IgE monoclonal antibodies, a class previously not used for cancer therapy, may present several potential advantages.^2^ In comparison to IgG and its Fcγ receptors, IgE has a high affinity for, and slower dissociation rate from, its cognate high-affinity Fcε receptor (FcεRI). This may result in longer retention of IgE on immune effector cells in tissues, including tumors. Furthermore, unlike IgG, IgE has no known inhibitory Fc receptors.^3^ Structurally, human IgE differs from human IgG by having an additional constant domain in its heavy chain, Cɛ2, replacing the hinge region found in IgG. This substitution gives IgE a more compact and rigid structure, significantly reducing the flexibility of its Fab arms relative to the Fc region and limiting conformational dynamics.^3^ Additionally, human IgE has seven glycosylation sites, including a high-mannose glycan at Asn394, while human IgG has a single glycosylation site at Asn297, which carries complex carbohydrates.^4^ These structural differences highlight the distinct conformational properties and most likely the functional characteristics of IgE compared to IgG. In a therapeutic setting, when recombinant IgE antibodies can be designed to recognize a tumor-associated antigen, several of the above attributes together may afford enhanced immune surveillance and superior effector cell stimulation against cancer cells.

Preclinical studies have shown that recombinant tumor antigen-specific IgE antibodies targeting several cancers can engender potent anti-tumor functions *in vitro* and *in vivo*, and drive induction of pro-inflammatory immunomodulatory conditions in the tumor microenvironment (TME).^2,5–11^ MOv18 IgE, the first-in-class therapeutic candidate to reach clinical testing, is a mouse/human chimeric monoclonal antibody that recognizes the tumor-associated antigen folate receptor alpha (FRα), expressed by several solid tumor types.^12^ In preclinical models, MOv18 IgE demonstrated superior anti-tumor efficacy, as compared to the IgG counterpart antibody, activation of TNF/MCP-1 signaling, and monocyte and macrophage recruitment and activation in tumors.^10,13–15^ These models demonstrated the unique mechanisms that IgE therapeutics may offer in activating immune cells to drive anti-tumoral functions.

Research Grade recombinant IgE antibodies have been generated and studied for several decades.^7,13,16–18^ Many studies report the production of high-purity antibodies with well-defined biophysical characteristics and functional profiles. However, recombinant IgE antibodies have not previously been generated at clinical grade for administration in humans.

The first-in-human Phase 1 clinical trial of the first-in-class MOv18 IgE (NCT02546921) has been successfully completed, demonstrating a good safety profile and early evidence of efficacy.^19^ This ground-breaking clinical trial necessitated the first-ever process development and manufacture of a recombinant IgE therapeutic candidate antibody at Good Manufacturing Practice (GMP) standard for use in humans. This endeavor required the transfer of the production of a recombinant IgE class antibody therapeutic candidate from preclinical Research Grade material, through process development, to large-scale production that was compliant with European Union GMP. This in turn necessitated the evaluation of whether GMP-grade material retained the biophysical and functional profile of the Research Grade antibody.

Here, we describe the characterization and functional assessment of the first-in-class recombinant therapeutic candidate MOv18 IgE during transfer from serum-rich to serum-free culture conditions, following low pH incubation used for viral inactivation, process development, and transfer to large-scale GMP production of clinical-grade antibody. Drug substances from each step of the process were evaluated for physio-chemical properties, as well as for retention of Fc receptor and antigen target binding, and Fc-mediated functional potency *in vitro*. Stability studies of MOv18 IgE preparations stored at 2–8°C in sterile bioprocess bags for up to 24 months were undertaken. In these evaluations, characteristics including substance pH, concentration, molecular weight, and purity were monitored. Levels of endotoxins, residual host cell DNA and protein, and other potential contaminants from the production process were assessed. Finally, we compared the Research Grade MOv18 IgE, used for preclinical studies, with the GMP grade bulk drug substance produced at large scale for clinical use, filled into glass vials, and used as the investigational medicinal product (IMP) for the Phase 1 clinical trial. Physio-chemical properties were assessed by SDS-PAGE (Sodium dodecyl sulfate polyacrylamide gel electrophoresis), SEC-HPLC (Size exclusion-high-performance liquid chromatography), and LC-MS (Liquid chromatography–mass spectrometry) glycan analysis. Fc-mediated functions were evaluated through mast cell degranulation and tumor cell killing assays. The potential for type I hypersensitivity was evaluated *ex vivo* using the basophil activation test (BAT) in cancer patients' whole blood.

## Results

### MOv18 IgE retains Fc receptor and target binding, and Fc-mediated functions, following transfer of production into serum-free culture conditions

**Figure 1.**
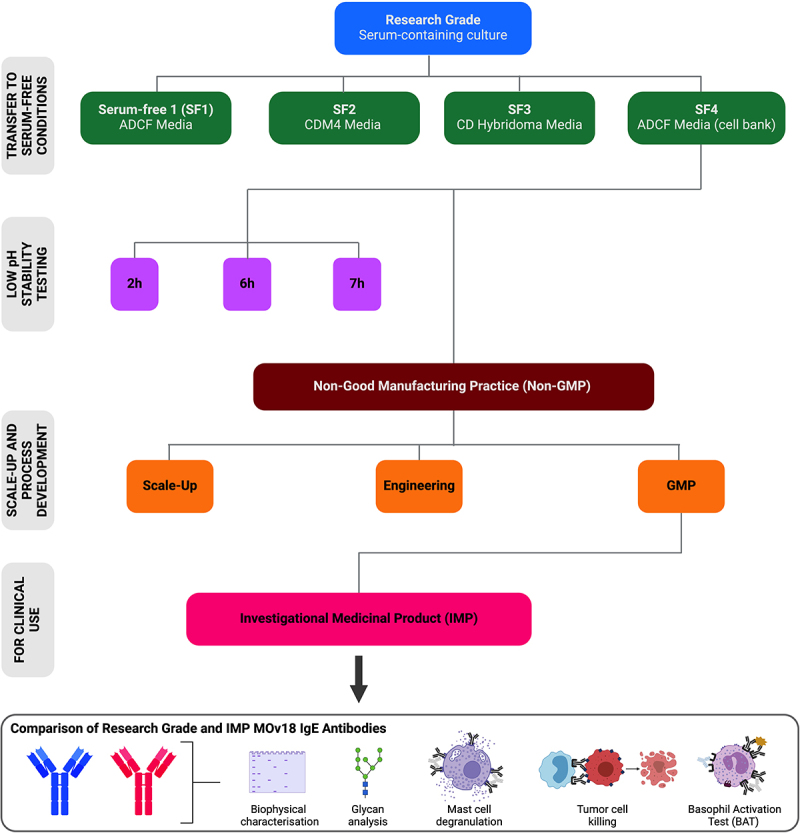
MOv18 IgE manufacturing process development pipeline. An overview of the process development pipeline and the batches of MOv18 IgE produced and tested. Firstly, the Sp2/0 mammalian expressing cells generated to produce research grade MOv18 IgE (blue) in serum-containing media, as previously reported, were transferred into four serum-free media conditions (green) and antibody preparations were evaluated. Mammalian cells adapted in serum-free conditions were used to: a) test antibody stability in low pH conditions (purple), and b) generate the non-gmp material (dark red). This was then used to generate the Scale-up, engineering and GMP batches (orange). The GMP batch was subsequently used as the IMP MOv18 IgE material in the phase I clinical trial of MOv18 IgE (pink). Research grade and IMP MOv18 IgE antibodies were comprehensibly compared for biophysical characteristics, glycan decoration, and Fc-mediated *in vitro* functions: mast cell degranulation upon cross-linking with polyclonal anti-IgE, tumor cell killing by fcɛRI-expressing human immune cells, and basophil activation test (BAT) (bottom box).

**Figure 2.**
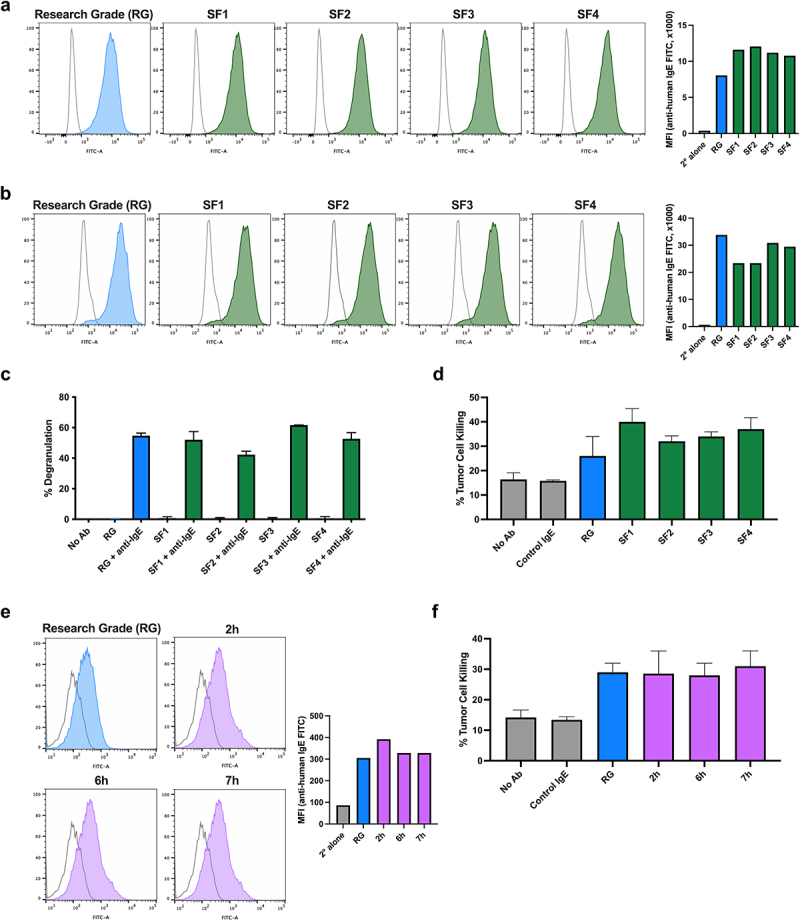
Characterization of MOv18 IgE following transfer to serum-free culture conditions and stability in low pH conditions. Comparability of research grade MOv18 IgE standard (RG; blue) with MOv18 IgE produced in serum-free medium conditions (SF1-SF4; green) was demonstrated by cell-surface binding to fcɛRI-expressing RBL-SX38 cells via the Fc domains (a) and Fab-mediated binding to fr⍺-expressing IGROV1 cancer cells (b). The antibody preparations also triggered comparable levels of RBL-SX38 mast cell degranulation in the presence, but not in the absence, of anti-IgE cross-linking antibody (c), and similar levels of tumor cell killing of IGROV1 tumor cells by U937 monocytic effector cells (d). Serum-free preparations of MOv18 IgE (purple) were tested following exposure to pH 3.5 between 2 to 7 hours and compared with research grade MOv18 IgE (blue) (e-f). Comparable cell surface binding to FRα-expressing CC531 cancer cells (e) and killing of FRα-expressing IGROV1 tumor cells by U937 monocytic effector cells (f) was demonstrated. 2° = secondary. Data presented as mean ± SD (*n* = 3) (c-f).

The original Research Grade chimeric MOv18 IgE antibody clone was produced in Sp2/0 cells, and all preclinical efficacy and mechanism of action studies of the antibody were generated with that material. To ensure that the antibody prepared for clinical testing is closely aligned with the structural and functional characteristics of Research Grade MOv18 IgE, a pipeline was established for the process development and evaluation of different batches of MOv18 IgE produced in the original Sp2/0 cells (Figure 1). The transfer of the MOv18 IgE-expressing Sp2/0 cells into serum-free conditions constituted the first step in this process. This was necessary to eliminate animal-derived components, ensure regulatory compliance, ensure safety, and maintain batch-to-batch consistency. Serum-free production also enhances scalability for clinical use and simplifies downstream purification. Antibody was generated in serum-free conditions at <5 L scale in shaker flasks using three commercially available media formulations (ADCF, CDM4, and CD Hybridoma) (SF1–3, Figure 1, green). An additional production run was carried out from a serum-free adapted cell bank, also in ADCF media (SF4, Figure 1, green). We next evaluated target and Fc-receptor binding, and Fc-mediated functions, of the antibodies generated under these serum-free culture conditions (SF1-SF4), in comparison with the Research Grade MOv18 IgE standard (blue). Research Grade IgE was produced in serum-containing cell culture conditions and used for preclinical evaluations^10,13,15,20^ (Figure 2).

Flow cytometric analysis of Research Grade and serum-free produced MOv18 IgE antibodies showed comparable cell-based binding characteristics to FcεRI-expressing rat basophilic leukemia RBL SX-38 cells (Figure 2a) and to tumor-associated antigen target, FRα-expressing IGROV1 cancer cells (Figure 2b). SDS-PAGE evaluation confirmed comparable molecular weight characteristics (Suplementary Figure S1). All IgE products triggered comparable levels of degranulation when cross-linked on the surface of FcεRI-expressing RBL SX-38 cells by polyclonal anti-IgE (Figure 2c). Lastly, all IgE preparations mediated comparable levels of tumor cell killing by human monocytic (U937) cells (Figure 2d).

These data suggest that MOv18 IgE generated in serum-free culture conditions retained the target antigen and Fc receptor binding, and Fc-mediated functionality and potency of the Research Grade IgE produced in serum-rich conditions.

### MOv18 IgE generated from cell culture scale-up and transfer to GMP production conditions retains target antigen-expressing cell binding and Fc-mediated functions

There is no precedent for the process development and GMP production of a recombinant IgE therapeutic antibody candidate. To allow for large scale GMP production of MOv18 IgE, a process development protocol was devised.

Firstly, we assessed the impact of low pH, used for viral inactivation, on antibody target-expressing cell recognition and Fc-mediated functions. Exposure to pH 3.5 for up to 7 h (Figure 1, purple) had minimal impact on MOv18 IgE binding to FRα-expressing cancer cells (CC531-FRα, Figure 2e). Similarly, irrespective of the incubation time at low pH (2 h to 7 h), all MOv18 IgE products showed comparable tumor cell killing potency when co-cultured with U937 human monocytic cells (Figure 2f). These findings suggest that exposure to low pH conditions for up to 7 h did not have a significant impact on MOv18 IgE activity.

**Figure 3.**
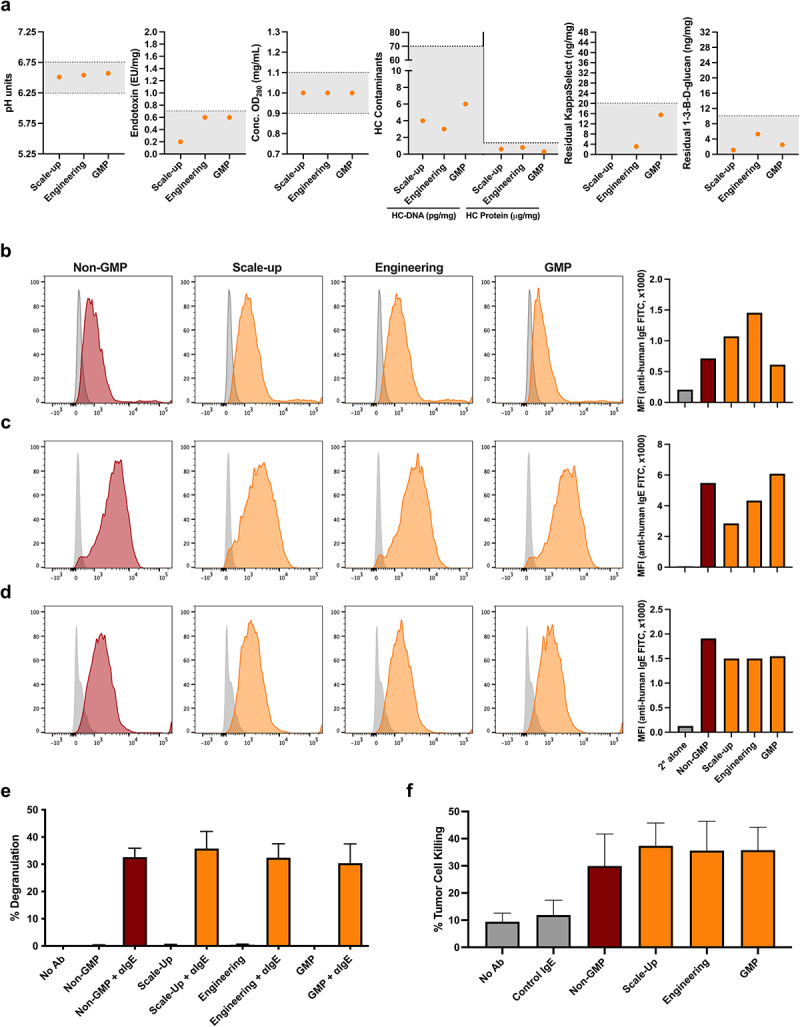
Batch and functional analysis of Scale-up, engineering and GMP preparations of MOv18 IgE. (a) Scale-up, engineering and GMP MOv18 IgE drug substances (orange) showed properties and levels of known contaminants within the following acceptance ranges and criteria (as indicated by horizontal dashed lines): pH (6.25–6.75), endotoxin levels (≤7.0 EU/mg), concentration (OD_280_) (0.90–1.10 mg/mL), residual host cell (HC) DNA and protein levels (≤70 pg/mg and ≤2.0 µg/mg, respectively), residual KappaSelect (≤20 ng/mg), and residual glucans (≤20 ng/mg) (a). Functional comparability of Scale-up, engineering and GMP MOv18 IgE drug substances (orange) compared with non-gmp MOv18 IgE (dark red) was demonstrated by: (b-d) cell-surface binding to FcεRI-expressing RBL-SX38 cells (b), and to FRα-expressing IGROV1 (c) and CC531 (d) cancer cells; (e-f) RBL-SX38 degranulation mediated by IgEs in the presence of anti-IgE cross-linking (e); tumor cell killing of FRα-expressing IGROV1 cancer cells by U937 monocytic cells (f), all in comparison to the non-gmp MOV18 IgE (dark red). 2° = secondary. Data presented as mean ± SD (*n* = 3) (e,f).

Next, to perform larger-scale production runs MOv18 IgE bulk drug substance was produced in a Scale-up run performed at 100 L scale. To confirm manufacturing parameters and specifications, a further non-GMP (Engineering) batch and a GMP batch were manufactured at 200 L scale. Batch analyses of larger scale MOv18 IgE preparations (Figure 1, orange) were compared with antibody generated from the ADCF media-adapted cell bank (non-GMP, Figure 1, dark red) (Figure 3a). Key properties, including pH, purity, and acceptable levels of known contaminants in manufacturing biological products were comparable among batches: the pH ranged from 6.51 to 6.57 (acceptable range: 6.25–6.75); endotoxin levels ranged from 0.2 to 0.6 endotoxin units (EU)/mg (acceptance criteria: ≤7.0 EU/mg); and concentration (OD_280_) was 1.00 mg/mL (acceptable range: 0.90–1.10 mg/mL) for all preparations. Residual host cell (HC) DNA and protein levels ranged from 3–6pg/mg to 0.3–0.8 µg/mg, respectively (acceptance criteria: ≤70 pg/mg and ≤2.0 µg/mg, respectively). Testing for any residual KappaSelect protein that may be shed from the affinity matrix used to purify MOv18 IgE from cell culture supernatants showed that residual levels ranged from 3.1 to 15.5 ng/mg (below the ≤20 ng/mg acceptance criteria) (Scale-up run was not tested). Furthermore, residual glucans, a potential contaminant in antibody production,^21,22^ were measured at 1.12–5.29 ng/mg (below the ≤20 ng/mg acceptance criteria). In summary, large-scale batches of MOv18 IgE drug substance, from Scale-up to GMP production, demonstrated expected properties and low levels of known manufacturing contaminants, all within acceptable ranges (Supplementary Materials; Specification for Final Testing Drug Substances section).

Having shown that MOv18 IgE retained functionality following transfer to a serum-free manufacturing process (Figures 2 and 3), we next tested the *in vitro* characteristics of non-GMP (dark red) alongside the Scale-up, Engineering, and GMP antibody batches (orange). Binding to FRα-expressing IGROV1 and CC531-FRα cancer cells and FcεRI-expressing RBL-SX38 cells was retained in all batches (Figures 3b-d). Degranulation of RBL-SX38 cells was triggered to a similar extent by all MOv18 IgE batches when cross-linked with anti-IgE antibody (Figure 3e). Furthermore, tumor cell killing by human U937 monocytic cells was mediated by all MOv18 IgE batches (Figure 3f).

These data suggest that MOv18 IgE retained target cell binding and Fc-mediated functions following transfer to production to large-scale and GMP processes.

### MOv18 IgE demonstrates an acceptable stability profile for up to 18 months

**Figure 4.**
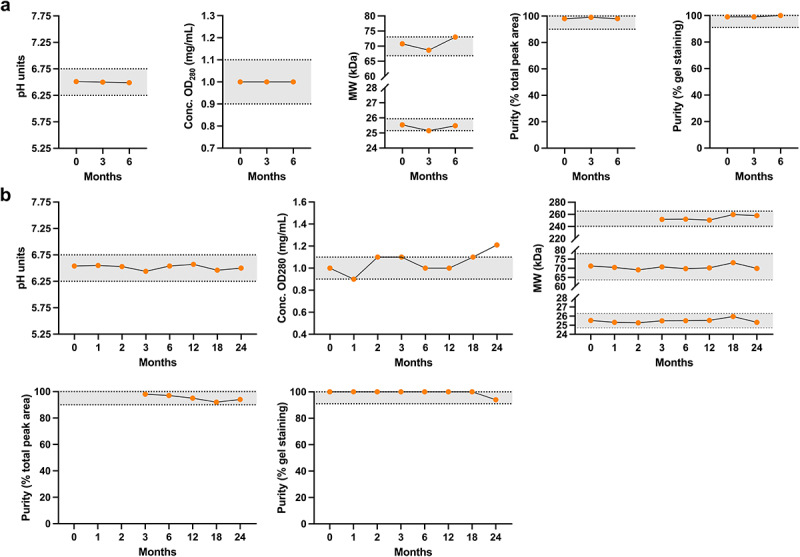
Stability of Scale-up, engineering and GMP preparations of MOv18 IgE. (a) Stability of the Scale-up MOv18 IgE preparation (orange) was demonstrated up to 6 months. (b) A formal stability study of the engineering batch showed that this antibody preparation was stable out to 24 months, except for the concentration (OD_280_) which showed an out of specification (OOS) measurement at 24 months. Specification ranges (indicated by horizontal dashed lines) were: pH (6.25–6.75); concentration (OD_280_) 0.90–1.10 mg/mL; reduced SDS-PAGE heavy chain (major band 1; 66.79–73.03 kDa, updated to 63.75–78.00 kDa for formal study of engineering batch), light chain (major band 2; 25.16–25.94 kDa, updated to 24.75–26.25 kDa for formal study of engineering batch) and total molecular weight determined by SEC-HPLC (240.25–265.25 kDa); purity determined by SEC-HPLC (≥90% of total peak area); and purity was determined by non-reduced SDS-PAGE (≥91% of gel staining).

An initial stability study was performed over the course of 6 months for the Scale-up batch of MOv18 IgE bulk drug substance in a sterile bioprocess bag, stored at 2–8°C (Figure 4a). The pH and concentration (OD_280_) of the stored antibody were consistent throughout the study: ranging from 6.49 to 6.51 (specification: 6.25–6.75) and maintained at 1.00 mg/mL (specification: 0.90–1.10 mg/mL), respectively. SDS-PAGE evaluation under reduced conditions showed consistent molecular weight ranges for the heavy chain (major band 1): 68.65–72.99 kDa (specification: 66.79–73.03 kDa) and light chain (major band 2): 25.15–25.54 kDa (specification: 25.16–25.94 kDa). Antibody purity determined by SDS-PAGE under non-reduced conditions was consistently 99–100% (specification: ≥91% of gel staining). Similarly, the antibody purity as determined by SEC-HPLC was measured consistently at 98–99% (specification ≥90% of total peak area).

A formal stability study of the Engineering batch of MOv18 IgE bulk drug substance, also stored in sterile bioprocess bags at 2–8°C, was performed. During this process, samples were taken at 0, 1, 2, 3, 6, 12, 18 and 24 months (Figure 4b). The product pH ranged from 6.44 to 6.57 (specification: 6.25–6.75), and antibody concentration (OD_280_) was consistent within the specification range of 0.90–1.10 mg/mL, except for a deviation to 1.21 mg/mL at 24 months. Thus, the shelf-life of the MOv18 IgE bulk drug substance was accepted to be at least 18 months when stored in sterile bioprocess bags at the recommended storage condition of 2–8°C. The MOv18 IgE Engineering batch, tested by SDS-PAGE under reduced conditions, showed consistent molecular weight ranges for the antibody heavy chain (major band 1): 69.11–73.08 kDa (specification: 63.75–78.00 kDa) and light chain (major band 2): 25.27–25.96 kDa (specification: 24.75–26.25 kDa). Total molecular weight determined by SEC-HPLC, was 250.37–257.91 kDa (specification: 240.25–265.25 kDa). Furthermore, the antibody purity as determined by non-reduced SDS-PAGE was consistently 100%, until 24 months in storage when the purity was measured at 94% (specification: ≥91% of gel staining). Purity determined by SEC-HPLC ranged between 92% and 98% (specification ≥90% of total peak area) (Figure 4b). Furthermore, the drug substance aseptically filled into the vials was tested for up to 84 months where 95.89% purity was measured using SDS-PAGE non-reduced, and 98.90% by HPLC (SEC) (Supplementary Figure S2).

Overall, the MOv18 IgE drug substance produced at large scale showed good stability for up to 18 months when stored in sterile bioprocess bags at 2–8°C.

### Assessment of MOv18 IgE IMP drug product

Bulk drug substance produced in the 200 L scale GMP batch (Figure 1, orange; see Supplementary Material for final manufacturing process and process control details) was allocated for clinical use. Following filling into glass vials, GMP batch material was determined as the drug product and IMP for the Phase 1 clinical trial of MOv18 IgE (Figure 1, pink). This final preparation was compared to the Research Grade MOv18 IgE (Figure 1, blue), which had been used for the preclinical studies of this agent.

**Figure 5.**
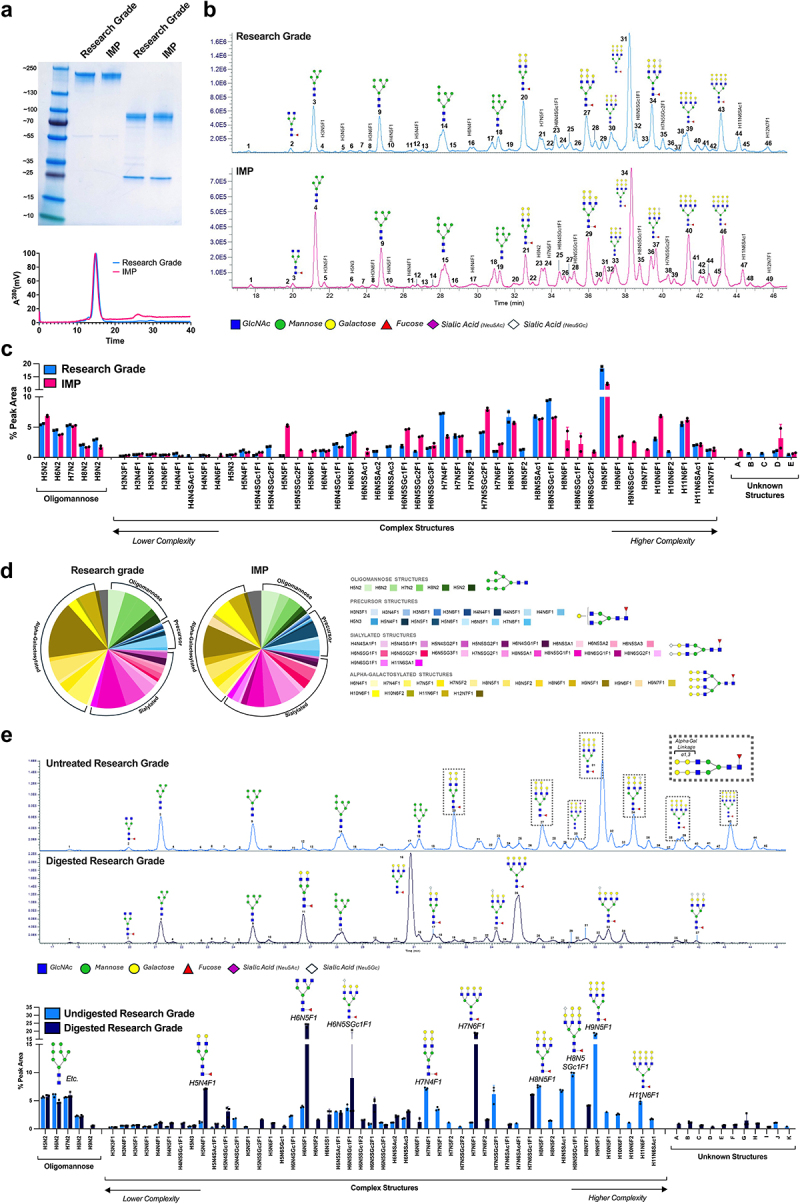
Physio-chemical analyses of MOv18 IMP prepared for the clinical trial. Comparability of research grade (blue) and IMP (pink) MOv18 IgE preparations was demonstrated by SDS-PAGE analysis (a top; non-reduced, left, reduced right), and SEC-HPLC (A bottom). (b-d) similar glycan profiles for research grade (top) and IMP (bottom) MOv18 IgE preparations are demonstrated in high-performance liquid chromatography with fluorescence detection (HPLC-FD) chromatograms. Suggested glycan structures assigned to the main peaks based on m/z masses and predicted monosaccharide compositions (b), and relative percentage areas of total detected HPLC chromatogram peaks containing individual glycan structures for released procainamide-labelled N-glycans (c and d). Monosaccharide compositions assigned to peaks area: *H*, hexose; *N*, N-acetylhexosamine; *SAc*, sialic acid (Neu5Ac); *SGc*, sialic acid (Neu5Gc); F, fucose. Unknown structures are as follows: A, H6N5SAc1SGc1or H5N5SGc2F1; B, H7N5SAc1 or H6N5SGc1F1; C, H7N5SAc1SGc1 or H6N5SGc2F1; D, H10N6SAc1 or H9N6SGcF1; E, H10N6SAc1SGc1 or H9N6SGc2F1. Supporting information can be found in supplementary figures 4–5 and observed m/z with predicted monosaccharide compositions and suggested structures for each labelled peak are shown in supplementary tables 1 and 2. (e) HPLC-fd chromatograms (e, top) and relative percentage areas of total detected HPLC chromatogram peaks (e, bottom) showing comparison of untreated research grade MOv18 IgE (top) and research grade MOv18 IgE treated with α-galactosidase. Suggested glycan structures assigned to main peaks are based on m/z masses and predicted monosaccharide compositions. These confirm decoration of MOv18 IgE with α-gal structures. Monosaccharide compositions assigned to peaks are: *H*, hexose; *N*, N-acetylhexosamine; *SAc*, sialic acid (Neu5Ac); *SGc*, sialic acid (Neu5Gc); F, fucose. Unknown structures are as follows: A, H5N5SAc1 or H4N5SGc1F1; B, H5N4SGc2F1 or H6N4SAc1SGc1; C, H6N6SAc1 or H5N6SGc1F1; D, H6N5SAc1SGc1 or H5N5SGc2F1; E, H7N5SAc1 or H6N5SGc1F1; F, H7N5SAc2 or H5N5SGc2F2; G, H7N5SAc1SGc1 or H6N5SGc2F1; H, H8N6SAc1SGc1 or H7N6SGc2F1; I, H9N7SAc1 or H8N7SGc1F1; J, H10N6SAc1or H9N6SGcF1; K, H10N6SAc1SGc1 or H9N6SGc2F1. Data presented as mean ± SD (*n* = 2) (C and E, bottom). Supporting information can be found in supplementary figures 6 and 7 and observed m/z with predicted monosaccharide compositions and suggested structures for each labelled peak are shown in supplementary tables 3 and 4.

Firstly, SDS-PAGE under non-reduced and reduced conditions (Figure 5a, top) showed comparable molecular weights for the Research Grade and IMP MOv18 IgE. Both preparations showed >95% purity by SEC-HPLC (Figure 5a, bottom). Treatment of MOv18 IgE with the deaminase enzyme, PNGase-F, resulted in a reduced molecular weight compared to untreated material (Supplementary Figure S3). Glycan analysis showed that the major predicted monosaccharide compositions and suggested glycan structures were largely similar between the Research Grade and IMP MOv18 IgE. Both MOv18 IgE antibody preparations showed mainly oligomannose, α-galactosylated and sialylated structures, with minor structural variations between antibodies, likely attributable to culture conditions (Figure 5b-d). The prediction of decoration by α-galactosidase (α-GAL), a glycan which is well reported for antibodies produced in Sp2/0 cells, was confirmed by the loss of these structures following specific digestion with ⍺-galactosidase in the Research Grade preparation (Figure 5e).

**Figure 6.**
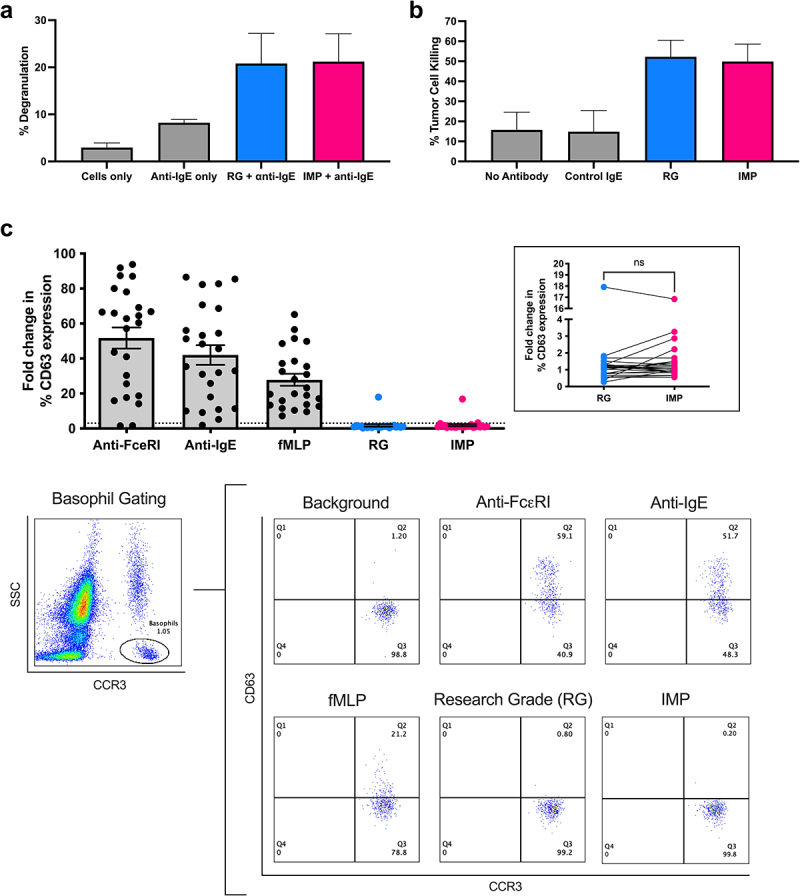
Functional and preliminary safety analyses of MOv18 IMP prepared for the clinical trial. Research grade (RG; blue) and IMP (pink) MOv18 IgE preparations triggered comparable levels of RBL-SX38 degranulation, in the presence of anti-IgE cross-linking (A; *n* = 3), and tumor cell killing of FRα-expressing IGROV1 cancer cells by U937 monocytic cells (B, *n* = 4). (C) With the exception of one patient sample, neither research grade (blue) and IMP (pink) MOv18 IgE preparations did not trigger CD63 upregulation by basophils in the basophil activation test (BAT) performed with whole unfractionated blood from ovarian cancer patients (C, top; *n* = 24; inset showing zoom of research grade and IMP IgE stimulation). (C, bottom): left: gating strategy to identify CCR3-PE^high^/SSC^low^ basophils in unfractionated blood; right: representative flow plots demonstrating cell surface CD63 expression levels under different conditions. Data presented as mean ± SD (A, B) or mean ± SEM (C). One-way ANOVA with šídák’s multiple comparisons test (A, B right). ns: not significant; *p ≤ 0.05; ***p ≤ 0.001.

Functional assessments demonstrated that Research Grade and IMP MOv18 IgE mediated comparable levels of degranulation by RBL SX-38 cells when cross-linked by anti-IgE (Figure 6A) and triggered comparable levels of tumor cell killing by U937 monocytic cells (Figure 6B).

Due to the known role of IgE antibodies in allergy and anaphylaxis, there is a perceived risk of anaphylaxis with intravenous administration of IgE therapeutics in humans. Furthermore, other clinically used monoclonal antibodies produced in Sp2/0 cells and decorated with α-GAL (as we confirmed for MOv18 IgE here, Supplementary Figures S4–7) are known to trigger type 1 hypersensitivity reactions in some sensitized individuals.^23–25^ We therefore applied the BAT, an assay used in the field of allergy. The BAT is conducted *ex vivo* with whole human blood and is designed to predict and monitor type I hypersensitivity, including to medicinal drugs and antibody therapeutics.^26–29^ Using the BAT, we assessed the potential for basophil activation, and thus for type I hypersensitivity, to both Research Grade and IMP MOv18 IgE antibodies. Activation of basophils in unfractionated whole blood from ovarian cancer patients was measured by the proportion of basophils showing upregulated CD63 surface expression. As expected, basophils showed upregulation of CD63 following *ex vivo* stimulation with known immune stimuli (anti-FcεRI, anti-IgE, and fMLP (*N*-Formylmethionyl-leucyl-phenylalanine)). However, neither the Research Grade, nor the IMP MOv18 IgE preparations engendered basophil activation above background, in any but one patient (Figure 6C). These data suggest that both Research Grade and IMP MOv18 IgE demonstrated similar functional characteristics.

In summary, physio-chemical, functional, and preliminary safety analyses of Research Grade and GMP-grade clinical MOv18 IgE material used as the IMP in the Phase 1 clinical trial, confirm that MOv18 IgE antibody preparations used in the non-clinical and clinical studies of this agent were comparable.

## Discussion

Established pipelines are widely used for the generation of clinical-grade IgG therapeutics. However, recombinant IgE antibodies have never before been generated at the clinical grade required for administration in humans.^7,10,13,16,18,30^ We report the characterization and functional assessment of the first-in-class recombinant therapeutic candidate MOv18 IgE through process development and GMP manufacture, which we establish for the first time for a recombinant IgE. We ascertain that all IgE products in the pipeline to IMP material retain the biophysical and functional characteristics of Research Grade MOv18 IgE.

We established a process development pipeline to 1) transfer the culture of MOv18 IgE-producing Sp2/0 mammalian cells from serum-containing to serum-free conditions, 2) assess the impact of low pH hold in the purification process required for viral inactivation, 3) increase production scale and achieve GMP-grade standards, 4) carry out antibody stability studies, and 5) compare the resulting material used as the IMP for the Phase 1 clinical trial of MOv18 IgE to the Research Grade antibody (Figure 1). Through this process, we demonstrate consistent biophysical and functional profiles of MOv18 IgE from Research Grade through the first ever process development and large-scale production of an IgE antibody, and ultimately in the GMP-grade material used as the IMP in the first-in-human, Phase 1 clinical trial of this first-in-class agent (NCT02546921).^19^

At each step of this pipeline, MOv18 IgE preparations were assessed for physio-chemical characteristics, and biological attributes, namely binding to cell-surface target antigen- and FcεRI-expressing cell models. Fc-mediated functionality, namely, RBL-SX38 cell degranulation following cross-linking with polyclonal anti-IgE antibody, and tumor cell killing by human FcεRI-expressing immune effector cells, were also evaluated. These properties, which have been previously demonstrated across numerous preclinical studies of MOv18 IgE using the Research Grade material,^10,13,15,20,31^ were shown to be retained irrespective of the transfer of MOv18 IgE-producing cell cultures into any of the three available standard serum-free media formulations studied (Figure 2). *In vitro* characteristics were retained by MOv18 IgE following exposure to low pH (Figure 2), in larger scale and GMP-grade productions (Figure 3), and were comparable between the Research Grade and IMP MOv18 IgE, used in the preclinical and clinical, studies of this agent, respectively (Figure 6).

We also evaluated the manufacturing parameters and specifications following the production scale-up of MOv18 IgE. These showed expected pH, purity, and concentration (OD_280_) properties and below threshold levels of known biological manufacturing contaminants, such as endotoxins, residual host cell DNA and protein, and residual glucans^21,22^ (Figure 4). Stability studies showed consistent results within acceptable specification ranges for parameters including pH, concentration (OD_280_), molecular weight, and purity over the course of 24 months (Figure 4).

Following the allocation of the bulk drug substance produced to GMP-grade for clinical use, the antibody was filled into glass vials and determined as the IMP for the Phase 1 clinical trial of MOv18 IgE. In assessing physio-chemical properties, we confirmed the comparability in molecular weight and purity between Research Grade and IMP MOv18 IgE preparations (Figure 5). Glycan analysis was also performed as it is of interest for IgE antibodies due to their high level of glycosylation compared to IgGs.^4^ In concordance, with prior studies of other IgE antibodies,^4,16,32^ key structures such as oligomannose, α-galactosylated, and sialylated, and their relative percentage areas of the chromatogram peaks of individual glycan structures, were comparable on Research Grade and IMP MOv18 IgE (Figure 5). Glycans have been reported to influence Fc receptor binding and functionality of IgEs.^32^ Therefore, similarities in the glycan profiles of Research Grade and IMP MOv18 IgE antibodies are concordant with our observations of comparable *in vitro* Fc-mediated functionality (Figure 6).

Stemming from the known roles of IgE in allergy and hypersensitivity reactions, a critical part for an anti-cancer IgE therapeutic candidate development is to address the perceived risk of anaphylaxis upon its administration in humans. The BAT is used in the field of allergy and in oncology to evaluate the potential for hypersensitivity to allergens, and to therapeutic drugs, including chemotherapies and monoclonal antibodies.^27,29,33,34^ The BAT is performed *ex vivo* in unfractionated whole blood, to account for any circulating factors able to cross-link IgE on the basophil surface and thus serve as mediators of type I hypersensitivity. These include soluble antigens and/or autoantibodies recognizing the antigen or binding to any part of the IgE structure in a patient's blood.^35–37^ Consistent with previous preclinical studies of MOv18 IgE and other tumor-targeting IgE antibodies, the BAT provided preliminary evidence of the absence of signs of type I hypersensitivity.^7–9,35^ In this study, we demonstrate that neither Research Grade nor IMP MOv18 IgE antibodies triggered *ex vivo* basophil activation. This was observed in all but one of the ovarian cancer patients whose blood samples were analyzed (Figure 6). This observation is particularly important, considering our results confirming the decoration of MOv18 IgE with α-GAL (Figure 5). Other clinically used monoclonal antibodies, such as cetuximab (IgG), that are expressed in Sp2/0 cells and therefore decorated with non-human glycan structures, such as α-GAL and Neu5Gc, have been associated with eliciting anti-drug antibodies and potential anaphylactic reactions in some patients.^38^ The BAT has been used to confirm basophil sensitivity in patients who reacted to cetuximab following treatment.^23–25^ In contrast, Chinese hamster ovary cells, which are commonly used to manufacture monoclonal antibodies, produce glycoproteins with glycosylation profiles more compatible and less likely to cause immune reactions.^38^ However, an important outcome from our data is that process development and GMP production have not significantly altered the glycan profile or the propensity of the Sp2/0-produced MOv18 IgE to trigger basophil activation when tested in cancer patient blood. This supports the suitability of the pipeline described herein for generating intact, functionally active, clinical-grade material, produced from the same cell line system, with comparable characteristics to the original Research Grade antibody. While such pipelines are widely used for the generation of IgG therapeutics, herein we report one such process established for the first time for a recombinant IgE therapeutic candidate.

The single ovarian cancer patient who showed sensitivity to both preparations of MOv18 IgE in the BAT went on to experience anaphylaxis when treated with IMP MOv18 IgE. Subsequently, the predicted sensitivity to MOv18 IgE in a BAT assay prior to treatment (at baseline) became an exclusion criterion for patient eligibility for the Phase 1 trial.^19^ Thus, the BAT may be able to predict the absence of hypersensitivity to IMP MOv18 IgE in the majority of individuals and may constitute a useful tool to predict type 1 hypersensitivity to MOv18 IgE, irrespective of the antibody preparation tested.

In conclusion, we achieved the successful process development, and the first ever large-scale and European Union GMP-compliant production of recombinant IgE for clinical use in the first-in-human clinical trial. We demonstrate that through this process, MOv18 IgE retains the biophysical and glycan profiles, as well as the cellular binding and functional characteristics of the extensively studied Research Grade MOv18 IgE. Furthermore, we confirm a preliminary assessment of safety, and the utility of the BAT to screen for patients potentially hypersensitive to MOv18 IgE administration and thus ineligible to receive the drug. This work supports the production of functionally active clinical grade recombinant IgE antibodies and paves the way for a new class of therapeutics that can be generated in a range of antigenic specificities and disease settings.

## Materials and methods

### Process development and MOv18 IgE evaluations

An overview of the process development and the batches of MOv18 IgE produced and tested herein is shown in Figure 1. Details of the final IMP manufacturing process and process controls are provided in the Supplementary Materials.

### Physio-chemical analyses

#### SDS-PAGE

Reduced and non-reduced SDS-PAGE were performed. For reduced conditions, 3 µg antibody was combined with a 1:4 ratio of dithiothreitol to 4X Laemmli Buffer, before incubation at 95°C for 5 min. For non-reduced conditions, 3 µg antibody was combined with 3 µl 4X Laemmli Buffer only. Antibody was pretreated with PNGase-F enzyme where indicated. Samples were loaded onto Mini-PROTEAN TGX Gels and run at 150 V for 45 min on a Mini-PROTEAN Tetra Vertical Electrophoresis Cell. Gels were incubated in InstantBlue Protein Stain for 1 h at room temperature under shaking conditions, then transferred to phosphate-buffered saline (PBS) overnight to remove excess stain prior to visualization. For stability studies, the gel scan was analyzed using pre-programmed software to determine the molecular weight against gel markers and calculate the relative percentage of each band within the lane.

#### SEC-HPLC

Samples, system suitability standards and a molecular weight standard solution were injected onto a size-exclusion column (Superdex 200 column previously equilibrated with PBS containing 0.1% (w/v) sodium azide). Material flowing through the column was detected via a UV/VIS detector at 280 nm. The results were analyzed using GPC software, reporting retention times, peak areas, and peak percentage. The molecular weight standard was used to generate a standard curve for calculation of sample molecular weight.

#### Hydrophilic interaction liquid chromatography HPLC for glycan analysis of IgE

Samples were as supplied with no clean up and dried down before use. For N-glycan release, samples were treated with PNGase-F and cleaned up prior to procainamide labeling. Following labels, samples underwent a second clean-up process and were concentrated prior to HPLC-FD-MS analysis as previously described.^32^ Samples were analyzed via Hydrophilic Interaction Liquid Chromatography (HILIC) HPLC using a Thermo Scientific Vanquish UHPLC instrument, with a BEH-Glycan 1.7 μm, 2.1 × 150 mm column (Waters) and a fluorescence detector (λex = 310 nm, λem = 370 nm) and controlled by Thermo Scientific Xcalibur software. MS analysis was performed using an Orbitrap Exploris 120 mass spectrometer, which was coupled directly after the UHPLC FD without splitting. HPLC-ESI-MS chromatogram analysis was performed using Xcalibur Data Acquisition and Interpretation Software version 4.3, and GlycoWorkbench software. Peak integration was performed using the PPD algorithm on Xcalibur Data Acquisition and Interpretation Software version 4.3, with manual peak integration used when necessary.

#### Quantification of galactose-α1,3-galactose levels

Samples were used as supplied with no clean-up. Galactose-α1,3-galactose linkages were released via α-galactosidase reagent (Sigma-Aldrich), then cleaned up prior to procainamide labeling. A separate untreated batch of samples were run concurrently for controls. Glycans were then processed and analyzed as described above. % Peak area was used to compare changes in glycan structure between treated and untreated samples.

### In vitro functional analyses

#### Cell culture

The U937 human monocytic cell line (ATCC CRL-1593.2), IGROV1 (CVCL_1304) ovarian cancer cell line, and human FRα-expressing CF531tFR rat colon carcinoma cell line (Cell Line Service^39^; kindly provided by Prof Silvana Canevari), were all cultured in RPMI 1640 medium, supplemented with 10% fetal calf serum, penicillin (5,000 U/ml), streptomycin (100 μg/ml) and maintained at 5% CO2, 37°C. The RBL-SX38 rat basophilic leukemia cells, which express both the native rat FcεRI and the human tetrameric form of FcεRI (kindly provided by Prof Jean-Pierre Kinet), were grown in the same conditions, with additional G418 (Gibco/Thermo Fisher Scientific) antibiotic selection.

#### Cell-surface binding

As previously described,^9,10^ 1 × 10^5^ cells were incubated with IgE antibodies at 5 µg/ml for 30 min at 4°C, followed by a wash with fluorescence-activated cell sorting (FACS) buffer (PBS pH 7.2 + 2% FBS). Next, 20 µg/ml anti-human IgE-FITC detection antibody (FI-3040; 2B Scientific) was added, and cells were incubated for another 30 min at 4°C and washed twice with FACS buffer before acquisition using a flow cytometer (Fortessa, Becton Dickinson).

#### Mast cell degranulation

MOv18 IgE-mediated degranulation of RBL-SX38 cells was performed as previously described.^9^ Briefly, RBL-SX38 cells were seeded at 1 × 10^4^ cells/well in 100 μl media 96-well round bottom plates (Nunc) and incubated at 37°C/5% CO_2_ overnight. The following day, the cells were sensitized with 200 ng/ml IgE or medium alone and incubated for 1 h at 37°C. Cells were washed 3 times in HBSS, 1% bovine serum albumin (BSA) (B4287; Sigma) and then cross-linked with 1.5 µg/ml polyclonal anti-IgE (A0094; Agilent Dako) for 1 h at 37°C. Control cells were lysed with Triton-X-100. All conditions were tested in triplicate. To quantify β-hexosaminidase release, 25 µl cell culture supernatant was diluted 1:1 with Hank’s balanced salt solution (HBSS; Life Technologies) and 1% BSA and transferred to wells of 96-well plates. Supernatant was incubated with 50 µl fluorogenic substrate (1 mmol/L 4-methylumbelliferyl N-acetyl-b-D-glucosaminide, 0.1% dimethyl sulfoxide, 200 mmol/L sodium citrate, pH 4.5) for 1 h in the dark at 37°C. Reactions were quenched using 100 µl 0.5 M Tris. Samples were measured following 350 nm excitation and 450 nm emission with a FLUOstar Omega Microplate Reader (BMG Labtech). Degranulation was expressed as a percentage of Triton X-100 release (100%).

#### Tumor cell killing

Tumor cell killing was evaluated using a three-color flow cytometric assay, as previously described.^9,10,40^ Tumor cells were pre-labeled one day prior to assay with carboxyfluorescein succinimidyl ester (CFSE) (Life Technologies). Briefly, tumor cells were detached with 0.5 M EDTA for up to 10 min, washed by centrifugation (1200 rpm for 5 min) in standard media and then in serum-free HBSS (Life Technologies). Per 1 × 10^6^ tumor cells, 0.75 μl of 0.5 μM CFSE was incubated for 10 min at 37°C. Cells were washed with RPMI 1640 + GLUTAMAX, then centrifuged as above for 5 min. The cell pellet was resuspended in RMPI 1640 + GLUTAMAX media and returned to culture overnight. The following day, CFSE labeled cells were detached, washed, counted, and resuspended to 1 × 10^6^ cells/ml. Control conditions of either no antibody or nonspecific isotype control IgE. Test samples were incubated with 5 μg/ml of MOv18 IgE. Following a wash with FACS buffer (PBS supplemented with 5% BSA), 100 μl human monocytic cells (U937s) effector cells and 100 μl tumor were added at an effector:target ratio of 3:1 to each tube with/without antibodies. Mixed cell co-cultures were incubated for 3 h at 37°C/5% CO2. After incubation, the cells were washed and incubated for 20 min at 4°C with 2 μg/ml APC-conjugated anti-CD89 antibody (354106; Biolegend) to label immune cells. Following a further wash, dead cells were labeled for 2 min at 4°C with DAPI (1:10000, Life Technologies) to label dead cells. Cells were washed and resuspended in FACS buffer and samples were acquired on a flow cytometer (FACSCanto II, Becton Dickinson). CFSE+/DAPI+ dead tumor cells (cytotoxicity) and CFSE+/APC+ cells (phagocytosis of CFSE+ tumor cells by APC+ effector cells) were gated. Total percentage of tumor cell killing was calculated as the sum of cytotoxicity and phagocytosis.

#### Basophil activation test

As previously described,^19,35^ the activation of basophils was analyzed by measurement of CD63 expression using the Flow2 CAST kit (FK-CCR; BÜHLMANN Laboratories AG), in accordance with the manufacturer’s instructions. About 100 μl unfractionated whole blood from ovarian cancer patients was incubated with 50 μl stimulation buffer (B-CCR-STB; Bühlmann Laboratories AG) and 100 μl of one of the following stimuli: anti-FcεRI (B-CCR-STCON; Bühlmann Laboratories AG), anti-IgE antibody (A0094; Agilent Dako), fMLP (B-CCR-FMLP; Bühlmann Laboratories AG), MOv18 IgE, or control IgE (at 3.5 μg/ml final concentration; in house). All samples were stained with 20 μl anti-CCR3-PE and anti-CD63-FITC staining cocktail (B-CCR-SR; Bühlmann Laboratories AG) and incubated at 37°C for 30 min in a 5% CO2 incubator. Following a further incubation with 2 ml red blood cell lysis (B-CCR-LYR; Bühlmann Laboratories AG) for 10 min at room temperature, samples were centrifuged at 500 G for 5 min, and cell pellets were resuspended in 150 μl acquisition buffer (B-CCR-WB; Bühlmann Laboratories AG). Samples were acquired on a flow cytometer (FACSCanto II, Becton Dickinson) whereby the CCR3-PE^high^/SSC^low^ basophil population was identified and analyzed for CD63 expression as a marker of activation.

## Supplementary Material

Bax et al Supplementary Materials Revised.docx

## Abbreviations

α-GAL: α-galactosidase
BAT: basophil activation test
BSA: bovine serum albumin
CFSE: carboxyfluorescein succinimidyl ester
EU: endotoxin units
FACS: fluorescence-activated cell sorting
FcεRI: high-affinity Fcε receptor
fMLP: *N*-Formylmethionyl-leucyl-phenylalanine
FRα: folate receptor alpha
GMP: Good Manufacturing Practice
HILIC: Hydrophilic Interaction Liquid Chromatography
IMP: Investigational Medicinal Product
HPLC-FD: high-performance liquid chromatography with fluorescence detection
LC-MS: Liquid chromatography – mass spectrometry
OOS: out of specification
PBS: phosphate-buffered saline
SDS-PAGE: Sodium dodecyl sulfate polyacrylamide gel electrophoresis
SEC-HPLC: Size exclusion-high-performance liquid chromatography
TME: tumor microenvironment
