# Supplementary material for: Physiochemical and functional evaluation of the first-in-class anti-cancer IgE antibody drug, MOv18, through process development and good manufacturing practice production
Source: MAbs. 2025 Jan 20;17(1):2451295. doi: 10.1080/19420862.2025.2451295 (PMC12952254; doi:10.1080/19420862.2025.2451295)
Supplement: Bax et al Supplementary Materials Revised.docx [file KMAB_A_2451295_SM0317.docx]

**SUPPLEMENTARY MATERIALS**

***Manufacturing Process and Process Controls***

The manufacturing process of MOv18 IgE IMP was formed of three main stages: Stage 1: Cell Expansion, Stage 2: Bioreactor Production Run, and Stage 3: Purification Process.

Stage 1 – Cell Expansion: A vial of the MOv18 IgE Master Cell Bank (MCB.12.01) was thawed and expanded sequentially in tissue culture and shaker flasks, followed by expansion in a 50L disposable stirred tank bioreactor. Cells were grown in serum-free ADCF-MAb media (Hyclone SH30349.02). Cells were passaged every 2 (seeding density 5 x 10^5^ viable cells/mL) or 3 (seeding density 2.5 x 10^5^ viable cells/mL) days. Cells underwent 6 - 7 passages to reach bioreactor inoculation density.

Stage 2 – Bioreactor Production Run: Cells from the 50L bioreactor expansion stage were transferred to a 250L single use bioreactor (SUB) for a 6 to 7-day production process, harvesting when cell viability was ≤ 60%. Cells were grown in ADCF-MAb medium. Process parameters such as dissolved oxygen (DO), pH and temperature are controlled to specified set points. The cell culture received 2.4 ± 0.2 L at 300 g/L glucose during the process to maximize cell growth and productivity. The final reactor volume was 240 ± 10L.

Stage 3 – Purification and Downstream Processing: The crude bioreactor harvest was filtered through a single depth filter to remove cells and cell debris, followed by a final filtration through a 0.2 μm filter. Following this, the clarified harvest underwent a viral inactivation step, to ensure the removal or inactivation of any potential viral contaminants. After viral inactivation, the material was further concentrated and purified through a series of affinity and ion-exchange chromatography steps, before a virus removal filtration, additional concentration, dia-filtration, and a final 0.2 μm filtration step into a sterile bioprocessing bag. Hold times have been derived from process development and the Engineering Batch, following analysis and characterisation. Packed columns undergo a defined sanitisation/rinse programme prior to and post use, followed by testing of the rinse water for conductivity/bioburden and endotoxin. Storage of columns had a defined schedule with set times for replacement of storage solution. Prior to use, columns underwent height equivalent to a theoretical plate (HETP) and asymmetry testing to ensure column performance.

**Batch analysis of manufacturing parameters and specifications and stability**

pH: The pH was measured using a calibrated pH-meter. Standards are used pre and post sample measurement to confirm accuracy of the result. Endotoxin: Endotoxin detection is conducted using Kinetic Chromogenic assay (Lonza) according to Ph. Eur. Protein Concentration (OD_280_): Determination of total protein concentration was performed by UV absorbance using the specific extinction coefficient of 1.4 (mg/mL)-1 cm at 1 cm path length. Residual Host Cell DNA: Host cell DNA was detected using a PCR method with primers against genomic NS0 Cells, another murine derived cell line. The kit is also suitable for use with SP2/0 cell lines. Extraction efficiency was determined using a positive control to ensure accuracy of the result. Residual Host Cell Protein**:** Residual host cell protein was determined by a commercially available ELISA kit (Cygnus Technologies) specific for SP2/0 cell derived proteins. Residual Kappa Select: Residual Kappa Select was determined by a commercially available ELISA kit (Cygnus Technologies) specific for Kappa Select. Residual 1,3-β-D-Glucan: Residual 1,3-β-D-Glucan is determined by a commercially available kit specific for 1,3- β-Glucans only. Molecular weight: Molecular weight was confirmed by non-reduced and reduced SDS-PAGE. Purity: Purity was assessed as percentage of total peak area of SEC-HPLC and percentage of gel staining of non-reduced SDS-PAGE.

***Specification for Final Testing Drug Substances***

The specification is intended for final testing of antibody HMBD-001 drug substance to be released for further manufacturing and use in a clinical phase I/lla study: Eudralex Annex 13 Investigational Medicinal Products;^1^ Eudralex Volume 4 Good Manufacturing Practice (GMP) guidelines;^1^ Monoclonal antibodies for human use, Ph. Eur. 01/2012:2031;^2^ ICHQ6B Specifications: test procedure and acceptance criteria for biotechnological/biological products;^3^ EMA/CHMP/BWP/157653/2007 Guideline on development, production, characterisation and specifications for monoclonal antibodies and related products;^4^ EMA/CHMP/SWP/28367/07 Rev. 1 Guidelines on strategies to identify and mitigate risks for first in human clinical trials with IMP EMA/CHMP/BWP/534898/2008 Rev. 1 Guideline on the requirements for quality documentation concerning biological investigational medicinal products in clinical trials;^5^ Products of recombinant DNA technology Ph. Eur. 01/2008:0784;^6^ and WHO Technical report No. 878.^7^

**Supplementary Figures and Tables**

**Supplementary Figure 1**

**
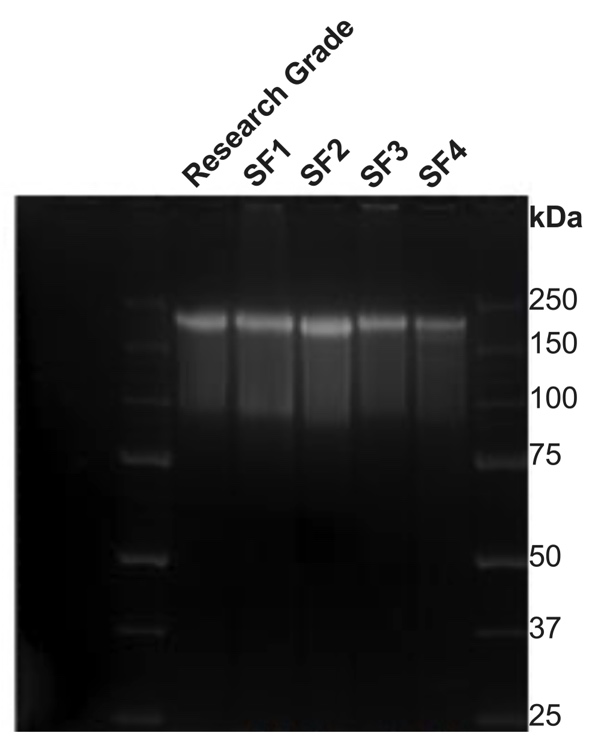
**

**Supplementary Figure 1.** Comparability of Research Grade MOv18 IgE standard with MOv18 IgE produced in serum-free medium conditions (SF1-SF4; green) was demonstrated by SDS-PAGE analysis.

**Supplementary Figure 2**

**
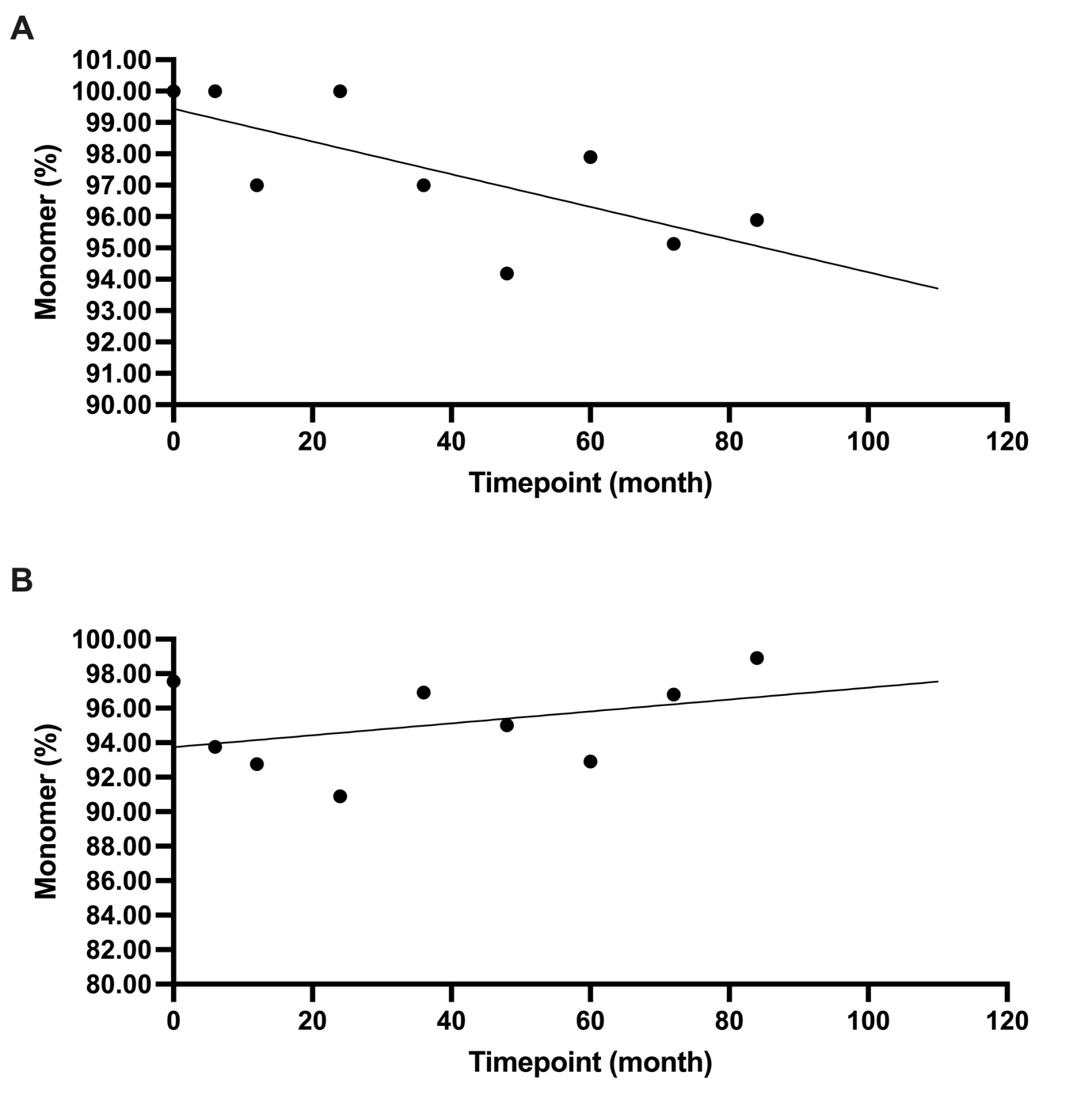
**

**Supplementary Figure 2.** (**A**) Trend of percentage of total staining accounted for by the specified bands of SDS-PAGE non-reduced over 84 months. (**B**) Trend of percentage monomer by HPLC-SEC of MAB.14.02 over 84 months.

**Supplementary Figure 3**


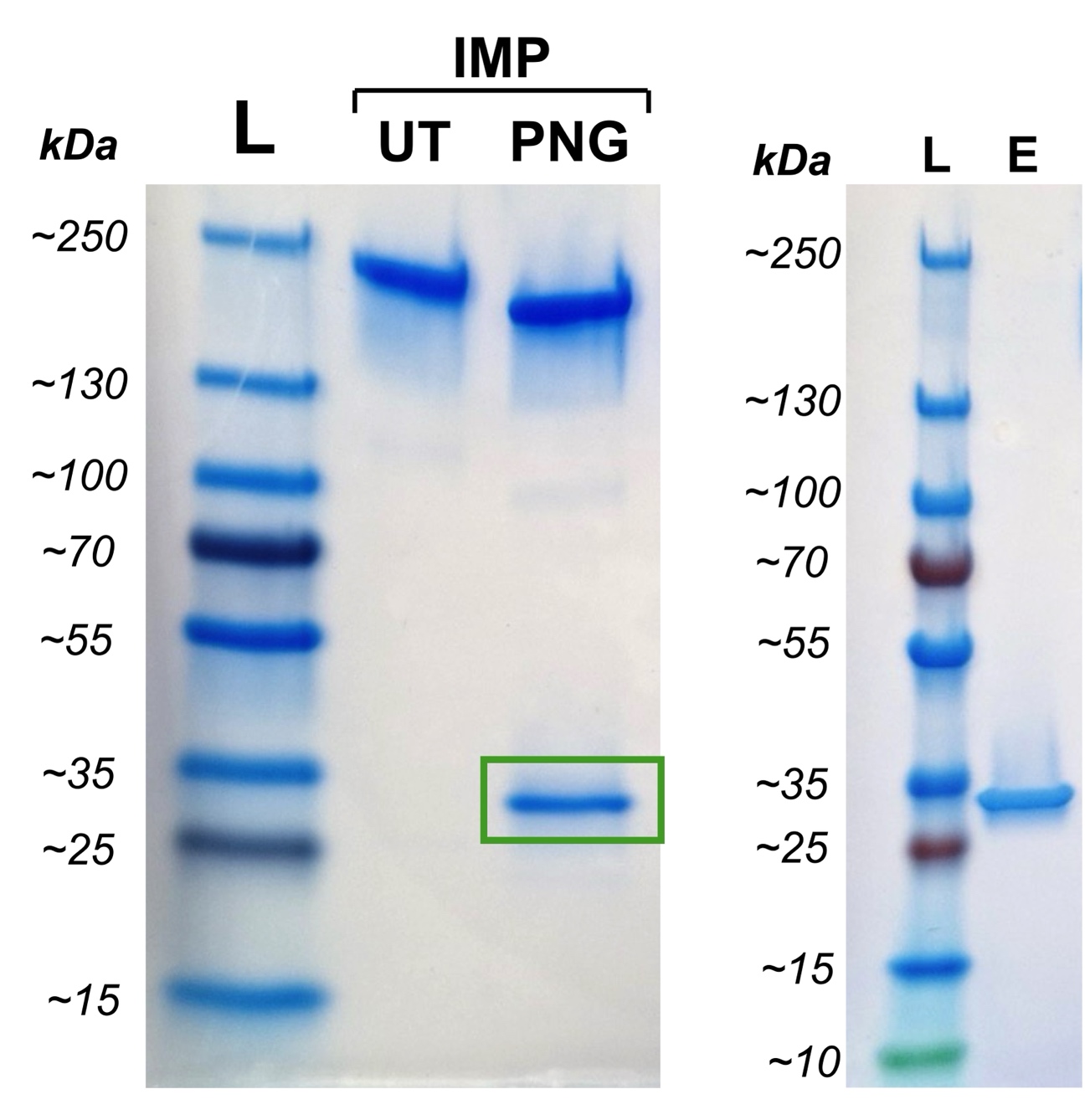


**Supplementary Figure 3**. Instant-Blue SDS-PAGE of MOv18 IgE IMP material treated with PNGase-F (left), and PNGase-F enzyme alone (right). L = molecular weight ladder; UT = untreated antibody; PNG = PNGase-F treated antibody; E = PNGase-F enzyme. Samples were run without clean-up, and green box indicates the presence of the PNGase-F enzyme in the treated material.

**Supplementary Figure 4**

**
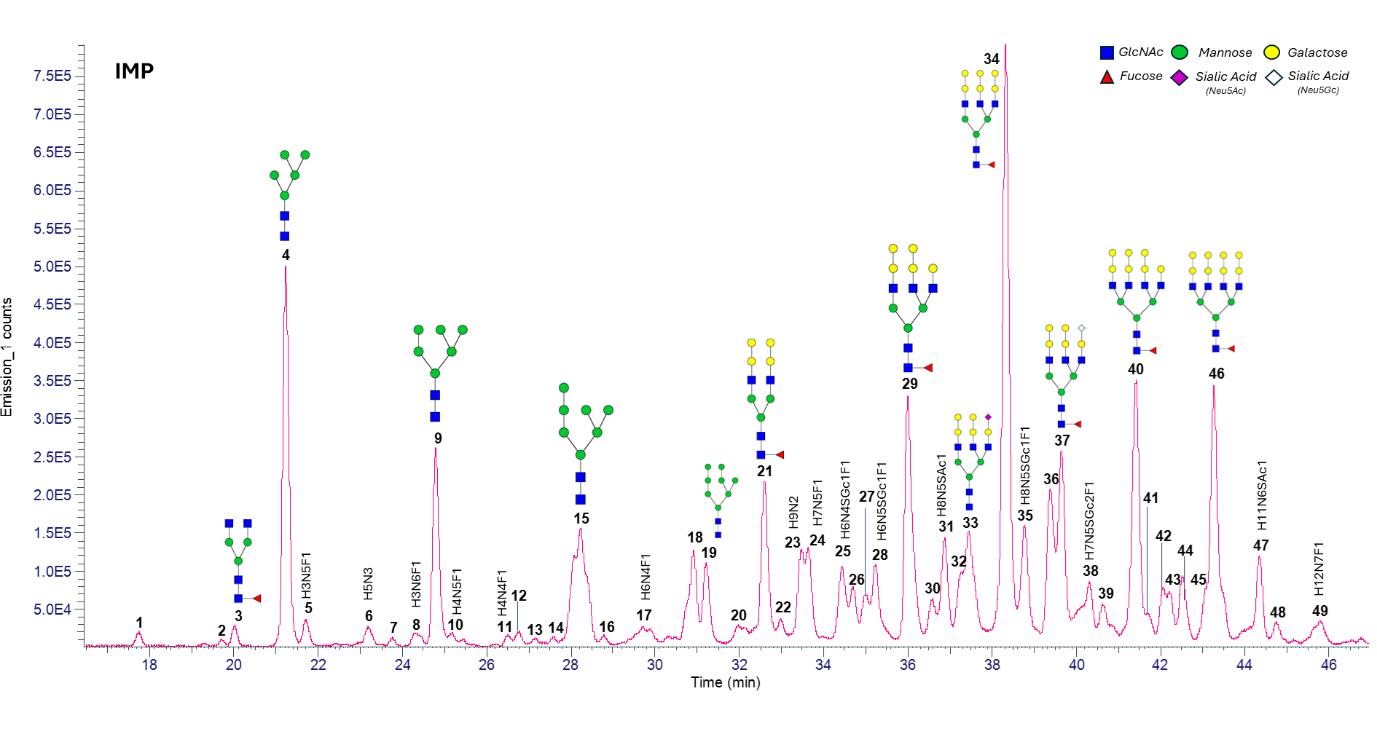
**

**Supplementary Figure 4**. HPLC-FD chromatogram for IMP MOv18 IgE [technical Replicate 1], with suggested glycan structures assigned to main peaks based on m/z masses and predicted monosaccharide compositions. Observed m/z with predicted monosaccharide compositions and suggested structures for each labelled peak are shown in Table 1, Replicate 1.

**Supplementary Figure 5**


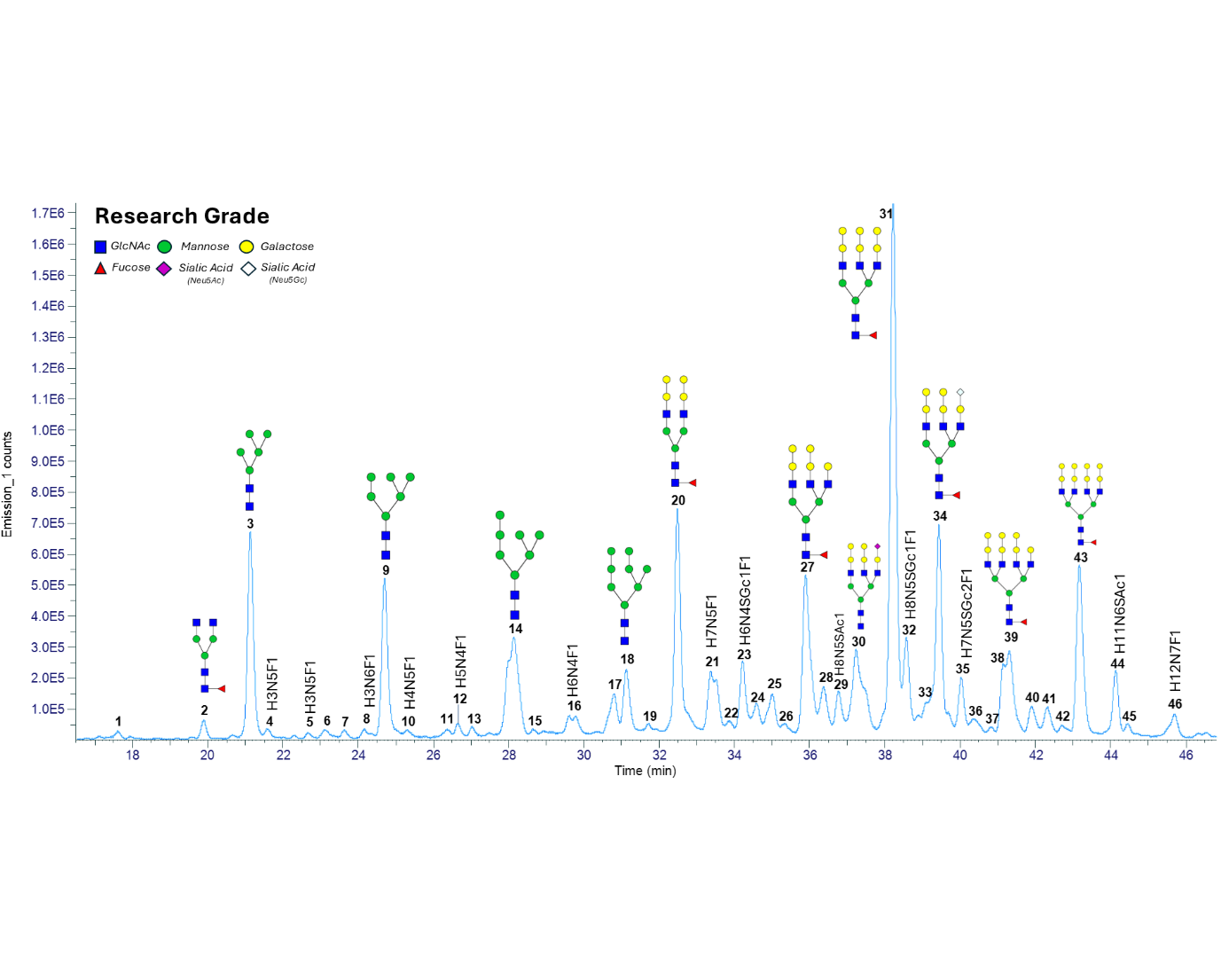


**Supplementary Figure 5**. HPLC-FD chromatogram for Research Grade MOv18 IgE [technical replicate 1], with suggested glycan structures assigned to main peaks based on m/z masses and predicted monosaccharide compositions. Observed m/z with predicted monosaccharide compositions and suggested structures for each labelled peak are shown in Table 2, Replicate 1.

**Supplementary Figure 6**

**
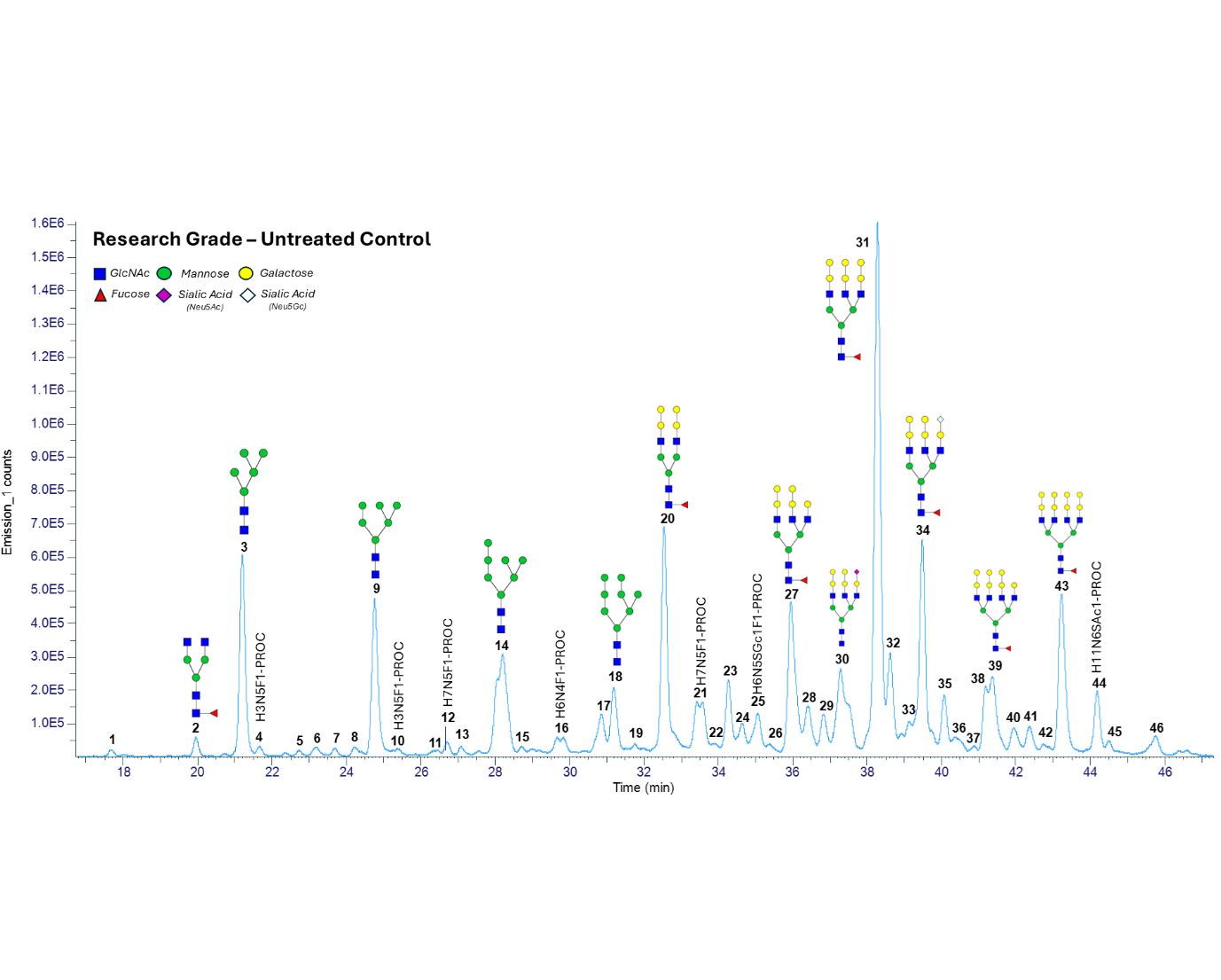
**

**Supplementary Figure 6**. HPLC-FD chromatogram for Research Grade MOv18 IgE AlphaGal Control [technical replicate 1], with suggested glycan structures assigned to main peaks based on m/z masses and predicted monosaccharide compositions. Observed m/z with predicted monosaccharide compositions and suggested structures for each labelled peak are shown in Table 3.

**Supplementary Figure 7**


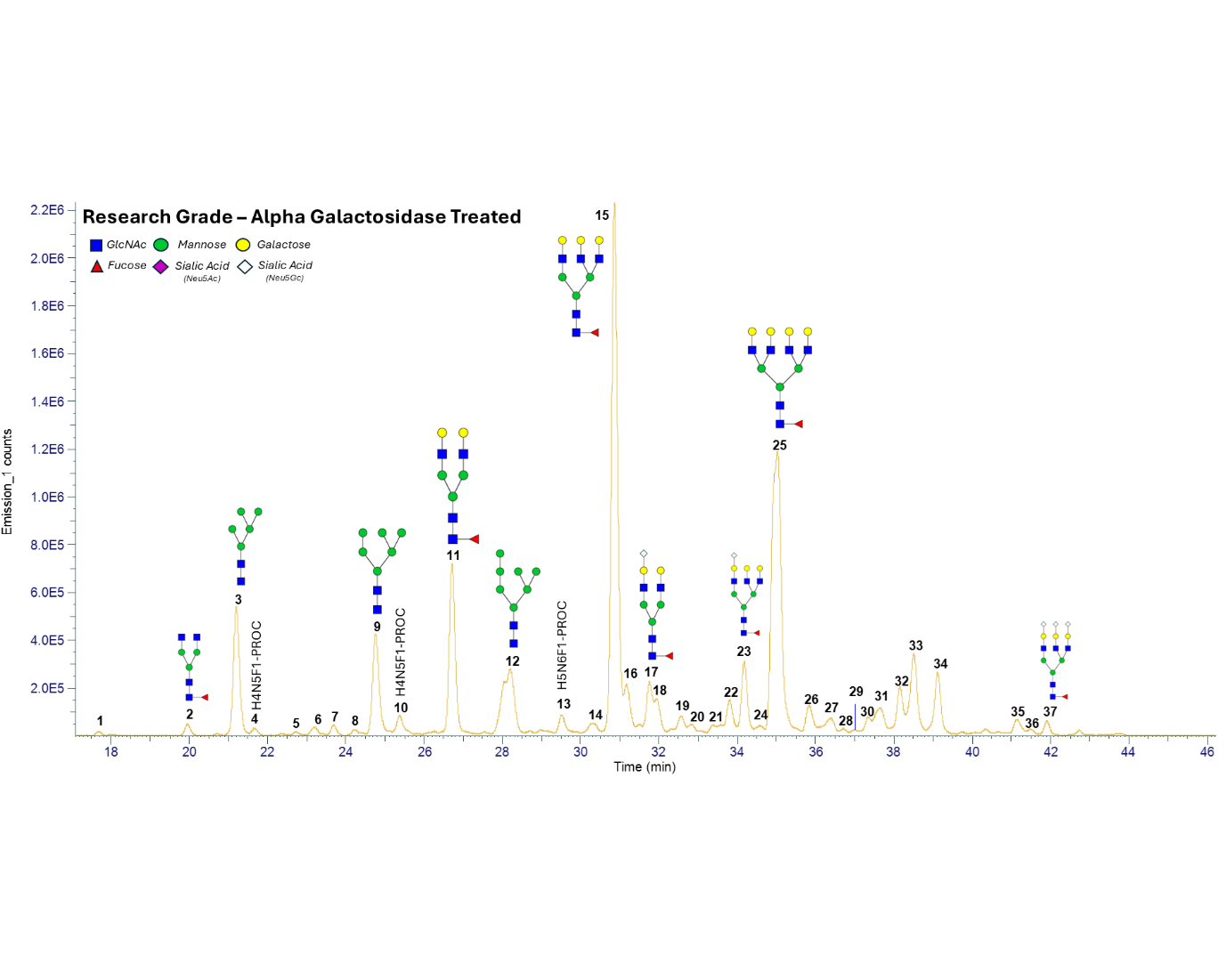


**Supplementary Figure 7**. HPLC-FD chromatogram for Research Grade MOv18 IgE - Alpha Galactosidase Treated [technical Replicate 1], with suggested glycan structures assigned to main peaks based on m/z masses and predicted monosaccharide compositions. Observed m/z with predicted monosaccharide compositions and suggested structures for each labelled peak are shown in Table 4.

**Supplementary Table 1**

Replicate 1

| **Peak #** | **Retention Time R1** | **% Peak Areas R1** | **Observed M/Z Value IMP-R1** | **Mass Error (PPM)** | **Calculated M/Z Value** | **Predicted Glycan Composition** |
| --- | --- | --- | --- | --- | --- | --- |
| **1** | 17.74 | 0.35 | 1479.6810^+1^ | 24.80 | 1479.6443^+1^ | H3N3F1-PROC |
| **2** | 19.72 | 0.16 | 841.8869^+2^ | 25.42 | 841.8655^+2^ | H3N4F1-PROC |
| **3** | 20.02 | 0.4 | 841.8859^+2^ | 24.23 | 841.8655^+2^ | H3N4F1-PROC |
| **4** | 21.23 | 6.98 | 727.8265^+2^ | 22.67 | 727.81^+2^ | H5N2-PROC |
| **5** | 21.71 | 0.58 | 943.4286^+2^ | 24.80 | 943.4052^+2^ | H3N5F1-PROC |
| **6** | 23.17 | 0.44 | 829.3707^+2^ | 25.32 | 829.3497^+2^ | H5N3-PROC |
| **7** | 23.76 | 0.2 | 922.9169^+2^ | 27.09 | 922.8919^+2^ | H4N4F1-PROC |
| **8** | 24.32 | 0.44 | 1044.9703^+2^ | 24.31 | 1044.9449^+2^ | H3N6F1-PROC |
| **9** | 24.79 | 3.85 | 808.8549^+2^ | 22.87 | 808.8364^+2^ | H6N2-PROC |
| **10** | 25.18 | 0.29 | 1024.4568^+2^ | 24.60 | 1024.4316^+2^ | H4N5F1-PROC |
| **11** | 26.51 | 0.21 | 1003.9452^+2^ | 26.80 | 1003.9183^+2^ | H5N4F1-PROC |
| **12** | 26.77 | 0.34 | 1003.9438^+2^ | 25.40 | 1003.9183^+2^ | H5N4F1-PROC |
| **13** | 27.17 | 0.25 | 1003.9439^+2^ | 25.50 | 1003.9183^+2^ | H5N4F1-PROC |
| **14** | 27.59 | 0.17 | 889.8868^+2^ | 26.97 | 889.8628^+2^ | H7N2-PROC |
|  |  |  | 1126.0006^+2^ | 26.02 | 1125.9713^+2^ | H4N6F1-PROC |
| **15** | 28.23 | 5.13 | 889.8837^+2^ | 23.49 | 889.8628^+2^ | H7N2-PROC |
|  |  |  | 1105.4835^+2^ | 23.07 | 1105.458^+2^ | H5N5F1-PROC |
| **16** | 28.79 | 0.28 | 1105.48620^+2^ | 25.51 | 1105.458^+2^ | H5N5F1-PROC |
| **17** | 29.71 | 1.15 | 1084.9718^+2^ | 24.98 | 1084.9447^+2^ | H6N4F1-PROC |
|  |  |  | 1207.0273^+2^ | 24.52 | 1206.9977^+2^ | H5N6F1-PROC |
| **18** | 30.91 | 2.42 | 1186.5105^+2^ | 22.00 | 1186.4844^+2^ | H6N5F1-PROC |
| **19** | 31.21 | 1.76 | 970.9133^+2^ | 24.82 | 970.8892^+2^ | H8N2-PROC |
|  |  |  | 1186.5133^+2^ | 24.36 | 1186.4844^+2^ | H6N5F1-PROC |
| **20** | 31.97 | 0.85 | 1157.4924^+2^ | 24.97 | 1157.4635^+2^ | H5N4SGc1F1-PROC |
|  |  |  | 1259.0343^+2^ | 24.70 | 1259.0032^+2^ | H6N5SAc1-PROC |
| **21** | 32.59 | 3.64 | 1165.9968^+2^ | 22.04 | 1165.9711^+2^ | H7N4F1-PROC |
| **22** | 32.98 | 0.53 | 1259.0354^+2^ | 25.58 | 1259.0032^+2^ | H6N5SAc1-PROC |
| **23** | 33.47 | 1.49 | 1051.9399^+2^ | 23.10 | 1051.9156^+2^ | H9N2-PROC |
|  |  |  | 1267.5395^+2^ | 22.64 | 1267.5108^+2^ | H7N5F1-PROC |
| **24** | 33.63 | 1.97 | 1267.5391^+2^ | 22.33 | 1267.5108^+2^ | H7N5F1-PROC |
| **25** | 34.44 | 1.7 | 1238.5190^+2^ | 23.50 | 1238.4899^+2^ | H6N4SGc1F1-PROC |
|  |  |  | 1340.0610^+2^ | 23.43 | 1340.0296^+2^ | H6N5SGc1F1-PROC |
| **26** | 34.70 | 1.09 | 1340.0624^+2^ | 24.48 | 1340.0296^+2^ | H6N5SGc1F1-PROC |
|  |  |  | 1369.0830^+2^ | 23.74 | 1369.0505^+2^ | H7N6F1-PROC |
| **27** | 35.00 | 1.08 | 1369.0834^+2^ | 24.03 | 1369.0505^+2^ | H7N6F1-PROC |
| **28** | 35.24 | 1.82 | 1340.0614^+2^ | 23.73 | 1340.0296^+2^ | H6N5SGc1F1-PROC |
| **29** | 35.10 | 5.83 | 1348.5680^+2^ | 22.84 | 1348.5372^+2^ | H8N5F1-PROC |
| **30** | 36.59 | 0.99 | 1421.0925^+2^ | 25.69 | 1421.056^+2^ | H8N5SAc1-PROC |
| **31** | 36.88 | 2.32 | 1421.0892^+2^ | 23.36 | 1421.056^+2^ | H8N5SAc1-PROC |
| **32** | 37.28 | 1.26 | 1450.1127^+2^ | 24.69 | 1450.0769^+2^ | H8N6F1-PROC |
|  |  |  | 1412.5826^+2^ | 24.28 | 1412.5483^+2^ | H6N5SAc1SGc1-PROC *OR* H5N5SGc2F1-PROC |
| **33** | 37.45 | 3.11 | 1421.0902^+2^ | 24.07 | 1421.056^+2^ | H8N5SAc1-PROC |
|  |  |  | 1450.1119^+2^ | 24.14 | 1450.0769^+2^ | H8N6F1-PROC |
| **34** | 38.32 | 12.59 | 1429.5967^+2^ | 23.15 | 1429.5636^+2^ | H9N5F1-PROC |
| **35** | 38.77 | 2.72 | 1502.1191^+2^ | 24.43 | 1502.0824^+2^ | H8N5SGc1F1-PROC |
| **36** | 39.38 | 3.55 | 1493.6110^+2^ | 24.30 | 1493.5747^+2^ | H6N5SGc2F1-PROC |
|  |  |  | 1531.1403^+2^ | 24.17 | 1531.1033^+2^ | H9N6F1-PROC |
| **37** | 39.64 | 3.94 | 1502.1168^+2^ | 22.90 | 1502.0824^+2^ | H8N5SGc1F1-PROC |
| **38** | 40.31 | 2.19 | 1574.6409^+2^ | 25.21 | 1574.6012^+2^ | H7N5SGc2F1-PROC |
|  |  |  | 1603.6648^+2^ | 26.63 | 1603.6221^+2^ | H8N6SGc1F1 |
| **39** | 40.63 | 1.31 | 1603.6643^+2^ | 26.32 | 1603.6221^+2^ | H8N6SGc1F1 |
|  |  |  | 1632.6855^+2^ | 26.03 | 1632.6430^+2^ | H9N7F1-PROC |
| **40** | 41.42 | 5.99 | 1574.6387^+2^ | 23.82 | 1574.6012^+2^ | H7N5SGc2F1-PROC |
|  |  |  | 1612.1681^+2^ | 23.82 | 1612.1297^+2^ | H10N6F1-PROC |
| **41** | 41.62 | 0.71 | 1612.1763^+2^ | 28.91 | 1612.1297^+2^ | H10N6F1-PROC |
|  |  |  | 1684.6965^+2^ | 28.49 | 1684.6485^+2^ | H10N6SAc1-PROC *OR*  H9N6SGcF1-PROC |
| **42** | 42.07 | 0.88 | 1684.6936^+2^ | 26.77 | 1684.6485^+2^ | H10N6SAc1-PROC *OR*  H9N6SGcF1-PROC |
| **43** | 42.21 | 0.85 | 1647.1655^+2^ | 27.68 | 1647.1199^+2^ | H6N5SGc3F1-PROC |
|  |  |  | 1684.6999^+2^ | 30.51 | 1684.6485^+2^ | H10N6SAc1-PROC *OR*  H9N6SGcF1-PROC |
| **44** | 42.51 | 1.71 | 1684.6968^+2^ | 28.67 | 1684.6485^+2^ | H10N6SAc1-PROC *OR*  H9N6SGcF1-PROC |
| **45** | 43.10 | 0.85 | 1647.1660^+2^ | 27.99 | 1647.1199^+2^ | H6N5SGc3F1-PROC |
|  |  |  | 1757.2158^+2^ | 27.60 | 1757.1673^+2^ | H8N6SGc2F1-PROC |
| **46** | 43.26 | 5.98 | 1693.1986^+2^ | 25.04 | 1693.1562^+2^ | H11N6F1-PROC |
| **47** | 44.35 | 1.93 | 1765.7248^+2^ | 28.26 | 1765.6749^+2^ | H11N6SAc1-PROC |
| **48** | 44.74 | 0.63 | 1838.2469^+2^ | 28.94 | 1838.1937^+2^ | H10N6SAc1SGc1-PROC *OR*  H9N6SGc2F1-PROC |
| **49** | 45.79 | 1.1 | 1875.7742^+2^ | 27.67 | 1875.7223^+2^ | H12N7F1-PROC |

Replicate 2

| **Peak #** | **Retention Time R1** | **% Peak Areas R1** | **Observed M/Z Value IMP-R1** | **Mass Error (PPM)** | **Calculated M/Z Value** | **Predicted Glycan Composition** |
| --- | --- | --- | --- | --- | --- | --- |
| **1** | 17.72 | 0.39 | 1479.6820^+1^ | 25.48 | 1479.6443^+1^ | H3N3F1-PROC |
| **2** | 19.72 | 0.2 | 841.8869^+2^ | 25.42 | 841.8655^+2^ | H3N4F1-PROC |
| **3** | 19.99 | 0.45 | 841.8868^+2^ | 25.30 | 841.8655^+2^ | H3N4F1-PROC |
| **4** | 21.22 | 6.71 | 727.8265^+2^ | 22.67 | 727.81^+2^ | H5N2-PROC |
| **5** | 21.71 | 0.6 | 943.4296^+2^ | 25.86 | 943.4052^+2^ | H3N5F1-PROC |
| **6** | 23.16 | 0.56 | 829.3714^+2^ | 26.17 | 829.3497^+2^ | H5N3-PROC |
| **7** | 23.75 | 0.25 | 922.9167^+2^ | 26.87 | 922.8919^+2^ | H4N4F1-PROC |
| **8** | 24.27 | 0.47 | 1044.9719^+2^ | 25.84 | 1044.9449^+2^ | H3N6F1-PROC |
| **9** | 24.79 | 3.72 | 808.85583^+2^ | 24.02 | 808.8364^+2^ | H6N2-PROC |
| **10** | 25.14 | 0.34 | 1024.4573^+2^ | 25.09 | 1024.4316^+2^ | H4N5F1-PROC |
| **11** | 26.49 | 0.25 | 1003.9448^+2^ | 26.40 | 1003.9183^+2^ | H5N4F1-PROC |
| **12** | 26.73 | 0.35 | 1003.9449^+2^ | 26.50 | 1003.9183^+2^ | H5N4F1-PROC |
| **13** | 27.10 | 0.29 | 1003.9446^+2^ | 26.20 | 1003.9183^+2^ | H5N4F1-PROC |
|  |  |  | 1126.0005^+2^ | 25.93 | 1125.9713^+2^ | H4N6F1-PROC |
| **14** | 27.59 | 0.25 | 889.88684^+2^ | 27.02 | 889.8628^+2^ | H7N2-PROC |
|  |  |  | 1126.0006^+2^ | 26.02 | 1125.9713^+2^ | H4N6F1-PROC |
| **15** | 28.22 | 4.84 | 889.88495^+2^ | 24.89 | 889.8628^+2^ | H7N2-PROC |
|  |  |  | 1105.4851^+2^ | 24.51 | 1105.458^+2^ | H5N5F1-PROC |
| **16** | 28.76 | 0.29 | 1105.4874^+2^ | 26.60 | 1105.458^+2^ | H5N5F1-PROC |
| **17** | 29.71 | 0.96 | 1084.9736^+2^ | 26.64 | 1084.9447^+2^ | H6N4F1-PROC |
|  |  |  | 1207.0293^+2^ | 26.18 | 1206.9977^+2^ | H5N6F1-PROC |
| **18** | 30.91 | 2.4 | 1186.5119^+2^ | 23.18 | 1186.4844^+2^ | H6N5F1-PROC |
| **19** | 31.20 | 1.65 | 970.91412^+2^ | 25.67 | 970.8892^+2^ | H8N2-PROC |
|  |  |  | 1186.5137^+2^ | 24.69 | 1186.4844^+2^ | H6N5F1-PROC |
| **20** | 31.98 | 0.86 | 1157.4936^+2^ | 26.01 | 1157.4635^+2^ | H5N4SGc1F1-PROC |
| **21** | 32.59 | 3.28 | 1165.9988^+2^ | 23.76 | 1165.9711^+2^ | H7N4F1-PROC |
| **22** | 32.97 | 0.65 | 1259.0351^+2^ | 25.34 | 1259.0032^+2^ | H6N5SAc1-PROC |
| **23** | 33.41 | 1.92 | 1051.9414^+2^ | 24.53 | 1051.9156^+2^ | H9N2-PROC |
|  |  |  | 1267.5419^+2^ | 24.54 | 1267.5108^+2^ | H7N5F1-PROC |
| **24** | 33.62 | 1.64 | 1267.5405^+2^ | 23.43 | 1267.5108^+2^ | H7N5F1-PROC |
| **25** | 34.43 | 1.75 | 1238.5200^+2^ | 24.30 | 1238.4899^+2^ | H6N4SGc1F1-PROC |
|  |  |  | 1340.0620^+2^ | 24.18 | 1340.0296^+2^ | H6N5SGc1F1-PROC |
| **26** | 34.69 | 1.2 | 1340.0629^+2^ | 24.85 | 1340.0296^+2^ | H6N5SGc1F1-PROC |
|  |  |  | 1369.0852^+2^ | 25.35 | 1369.0505^+2^ | H7N6F1-PROC |
| **27** | 34.99 | 1.09 | 1369.0857^+2^ | 25.71 | 1369.0505^+2^ | H7N6F1-PROC |
| **28** | 35.22 | 1.78 | 1340.0639^+2^ | 25.60 | 1340.0296^+2^ | H6N5SGc1F1-PROC |
| **29** | 35.99 | 5.58 | 1348.5679^+2^ | 22.77 | 1348.5372^+2^ | H8N5F1-PROC |
| **30** | 36.56 | 1.16 | 1421.0924^+2^ | 25.61 | 1421.056^+2^ | H8N5SAc1-PROC |
| **31** | 36.86 | 2.14 | 1421.0906^+2^ | 24.35 | 1421.056^+2^ | H8N5SAc1-PROC |
| **32** | 37.29 | 1.3 | 1450.1145^+2^ | 25.93 | 1450.0769^+2^ | H8N6F1-PROC |
|  |  |  | 1412.5849^+2^ | 25.91 | 1412.5483^+2^ | H6N5SAc1SGc1-PROC *OR* H5N5SGc2F1-PROC |
| **33** | 37.44 | 2.99 | 1421.0947^+2^ | 27.23 | 1421.056^+2^ | H8N5SAc1-PROC |
| **34** | 38.31 | 11.98 | 1429.5936^+2^ | 20.99 | 1429.5636^+2^ | H9N5F1-PROC |
| **35** | 38.77 | 2.66 | 1502.1201^+2^ | 25.10 | 1502.0824^+2^ | H8N5SGc1F1-PROC |
| **36** | 39.37 | 3.39 | 1493.6124^+2^ | 25.24 | 1493.5747^+2^ | H6N5SGc2F1-PROC |
|  |  |  | 1531.1417^+2^ | 25.08 | 1531.1033^+2^ | H9N6F1-PROC |
| **37** | 39.64 | 3.84 | 1502.1199^+2^ | 24.97 | 1502.0824^+2^ | H8N5SGc1F1-PROC |
| **38** | 40.29 | 1.57 | 1574.6460^+2^ | 28.45 | 1574.6012^+2^ | H7N5SGc2F1-PROC |
| **39** | 40.63 | 0.98 | 1603.6681^+2^ | 28.69 | 1603.6221^+2^ | H8N6SGc1F1 |
| **40** | 41.41 | 6.17 | 1574.6426^+2^ | 26.29 | 1574.6012^+2^ | H7N5SGc2F1-PROC |
|  |  |  | 1612.1719^+2^ | 26.18 | 1612.1297^+2^ | H10N6F1-PROC |
| **41** | 41.69 | 0.8 | 1612.1749^+2^ | 28.04 | 1612.1297^+2^ | H10N6F1-PROC |
|  |  |  | 1684.6968^+2^ | 28.67 | 1684.6485^+2^ | H10N6SAc1-PROC *OR*  H9N6SGcF1-PROC |
| **42** | 42.06 | 1.05 | 1684.6984^+2^ | 29.62 | 1684.6485^+2^ | H10N6SAc1-PROC *OR*  H9N6SGcF1-PROC |
| **43** | 42.20 | 1.25 | 1647.1664^+2^ | 28.23 | 1647.1199^+2^ | H6N5SGc3F1-PROC |
|  |  |  | 1684.6970^+2^ | 28.79 | 1684.6485^+2^ | H10N6SAc1-PROC *OR*  H9N6SGcF1-PROC |
| **44** | 42.51 | 1.72 | 1684.6969^+2^ | 28.73 | 1684.6485^+2^ | H10N6SAc1-PROC *OR*  H9N6SGcF1-PROC |
| **45** | 43.11 | 1.1 | 1647.1647^+2^ | 27.20 | 1647.1199^+2^ | H6N5SGc3F1-PROC |
|  |  |  | 1757.2129^+2^ | 25.95 | 1757.1673^+2^ | H8N6SGc2F1-PROC |
| **46** | 43.26 | 6.46 | 1693.1999^+2^ | 25.81 | 1693.1562^+2^ | H11N6F1-PROC |
| **47** | 44.33 | 2.31 | 1765.7234^+2^ | 27.47 | 1765.6749^+2^ | H11N6SAc1-PROC |
| **48** | 44.76 | 0.79 | 1838.2436^+2^ | 27.15 | 1838.1937^+2^ | H10N6SAc1SGc1-PROC *OR*  H9N6SGc2F1-PROC |
| **49** | 45.81 | 1.58 | 1875.7712^+2^ | 26.07 | 1875.7223^+2^ | H12N7F1-PROC |

**Supplementary Table 1.** Comparison of IMP MOv18 IgE, technical Replicate 1 shown in red and replicate 2 shown in blue. Shown are predicted monosaccharide compositions and suggested glycan structures based on observed MS m/z masses. H = hexose; N = N-acetylhexosamine; F = fucose; SGc = sialic acid (Neu5Gc); Sac = sialic acid (Neu5Ac).

**Supplementary Table 2**

Replicate 1

| **Peak #** | **Retention Time**  **R1** | **% Peak Areas** | **Observed M/Z Value**  **R1** | **Mass Error (PPM)** | **Calculated M/Z Value** | **Predicted Glycan Composition** |
| --- | --- | --- | --- | --- | --- | --- |
| **1** | 17.61 | 0.26 | 1479.6578^+1^ | 9.12 | 1479.6443^+1^ | H3N3F1-PROC |
| **2** | 19.90 | 0.47 | 841.8659^+2^ | 0.48 | 841.8655^+2^ | H3N4F1-PROC |
| **3** | 21.14 | 5.39 | 727.8095^+2^ | -0.69 | 727.81^+2^ | H5N2-PROC |
| **4** | 21.61 | 0.28 | 943.4039^+2^ | -1.38 | 943.4052^+2^ | H3N5F1-PROC |
| **5** | 22.65 | 0.18 | 943.4048^+2^ | -0.42 | 943.4052^+2^ | H3N5F1-PROC |
| **6** | 23.13 | 0.44 | 829.3544^+2^ | 5.67 | 829.3497^+2^ | H5N3-PROC |
|  |  |  | 922.8932^+2^ | 1.41 | 922.8919^+2^ | H4N4F1-PROC |
| **7** | 23.63 | 0.28 | 922.8942^+2^ | 2.49 | 922.8919^+2^ | H4N4F1-PROC |
| **8** | 24.16 | 0.41 | 1044.9511^+2^ | 5.93 | 1044.9449^+2^ | H3N6F1-PROC |
| **9** | 24.70 | 4.57 | 808.8364^+2^ | 0.00 | 808.8364^+2^ | H6N2-PROC |
| **10** | 25.30 | 0.39 | 1024.4263^+2^ | -5.17 | 1024.4316^+2^ | H4N5F1-PROC |
| **11** | 26.37 | 0.42 | 1003.9234^+2^ | 5.08 | 1003.9183^+2^ | H5N4F1-PROC |
| **12** | 26.64 | 0.49 | 1003.9200^+2^ | 1.69 | 1003.9183^+2^ | H5N4F1-PROC |
| **13** | 27.02 | 0.34 | 1003.9231^+2^ | 4.78 | 1003.9183^+2^ | H5N4F1-PROC |
| **14** | 28.14 | 5.24 | 889.8646^+2^ | 2.02 | 889.8628^+2^ | H7N2-PROC |
| **15** | 28.65 | 0.31 | 1105.4626^+2^ | 4.16 | 1105.458^+2^ | H5N5F1-PROC |
| **16** | 29.62 | 1.22 | 1084.9517^+2^ | 6.45 | 1084.9447^+2^ | H6N4F1-PROC |
| **17** | 30.79 | 1.6 | 1186.4924^+2^ | 6.74 | 1186.4844^+2^ | H6N5F1-PROC |
| **18** | 31.13 | 2.04 | 970.8889^+2^ | -0.31 | 970.8892^+2^ | H8N2-PROC |
|  |  |  | 1186.4873^+2^ | 2.44 | 1186.4844^+2^ | H6N5F1-PROC |
| **19** | 31.72 | 0.51 | 1157.4661^+2^ | 2.25 | 1157.4635^+2^ | H5N4SGc1F1-PROC |
| **20** | 32.48 | 7.32 | 1165.9786^+2^ | 6.43 | 1165.9711^+2^ | H7N4F1-PROC |
| **21** | 33.37 | 3.07 | 1051.9169^+2^ | 1.24 | 1051.9156^+2^ | H9N2-PROC |
|  |  |  | 1267.5199^+2^ | 7.18 | 1267.5108^+2^ | H7N5F1-PROC |
| **22** | 33.87 | 0.69 | 1267.5204^+2^ | 7.57 | 1267.5108^+2^ | H7N5F1-PROC |
| **23** | 34.22 | 2.32 | 1238.4988^+2^ | 7.19 | 1238.4899^+2^ | H6N4SGc1F1-PROC |
| **24** | 34.58 | 1.02 | 1340.5413^+2^ | 1.12 | 1340.5398^+2^ | H7N5F2-PROC |
|  |  |  | 1369.0546^+2^ | 2.99 | 1369.0505^+2^ | H7N6F1-PROC |
|  |  |  | 1404.5565^+2^ | 3.99 | 1404.5509^+2^ | H6N5SAc2-PROC |
| **25** | 35.00 | 1.73 | 1340.0391^+2^ | 7.09 | 1340.0296^+2^ | H6N5SGc1F1-PROC |
| **26** | 35.32 | 0.67 | 1340.0425^+2^ | 9.63 | 1340.0296^+2^ | H7N5SAc1-PROC *OR* H6N5SGc1F1-PROC |
| **27** | 35.89 | 5.83 | 1348.5436^+2^ | 4.75 | 1348.5372^+2^ | H8N5F1-PROC |
| **28** | 36.36 | 1.85 | 1311.0386^+2^ | 22.88 | 1311.0086^+2^ | H5N4SGc2F1-PROC |
|  |  |  | 1421.0646^+2^ | -352.85 | 1421.5662^+2^ | H8N5SAc1-PROC |
|  |  |  | 1550.0985^+2^ | -0.06 | 1550.0986^+2^ | H6N5SAc3-PROC |
| **29** | 36.76 | 1.26 | 1421.5730^+2^ | 4.78 | 1421.5662^+2^ | H8N5F2-PROC |
| **30** | 37.23 | 5.04 | 1421.0610^+2^ | -355.38 | 1421.5662^+2^ | H8N5SAc1-PROC |
| **31** | 38.22 | 14.96 | 1429.5675^+2^ | 2.73 | 1429.5636^+2^ | H9N5F1-PROC |
| **32** | 38.56 | 2.58 | 1429.5679^+2^ | 3.01 | 1429.5636^+2^ | H9N5F1-PROC |
|  |  |  | 1502.0813^+2^ | -0.73 | 1502.0824^+2^ | H8N5SGc1F1-PROC |
| **33** | 39.18 | 1.02 | 1493.5762^+2^ | 1.00 | 1493.5747^+2^ | H7N5SAc1SGc1-PROC *OR* H6N5SGc2F1-PROC |
| **34** | 39.43 | 6.8 | 1502.0808^+2^ | -1.07 | 1502.0824^+2^ | H8N5SGc1F1-PROC |
| **35** | 40.03 | 1.63 | 1574.5953^+2^ | -3.75 | 1574.6012^+2^ | H7N5SGc2F1-PROC |
| **36** | 40.37 | 0.88 | 1574.6237^+2^ | 14.29 | 1574.6012^+2^ | H7N5SGc2F1-PROC |
| **37** | *40.83* | *0.37* | *Not detected* | | | |
| **38** | 41.14 | 1.69 | 1574.5963^+2^ | -3.11 | 1574.6012^+2^ | H7N5SGc2F1-PROC |
| **39** | 41.31 | 2.9 | 1612.1228^+2^ | -4.28 | 1612.1297^+2^ | H10N6F1-PROC |
| **40** | 41.89 | 0.98 | 1647.1219^+2^ | 1.21 | 1647.1199^+2^ | H6N5SGc3F1-PROC |
|  |  |  | 1685.1592^+2^ | 0.30 | 1685.1587^+2^ | H10N6F2-PROC |
| **41** | 42.33 | 0.92 | 1684.6398^+2^ | -5.16 | 1684.6485^+2^ | H10N6SAc1-PROC *OR*  H9N6SGcF1-PROC |
| **42** | 42.69 | 0.52 | 1647.1398^+2^ | 12.08 | 1647.1199^+2^ | H6N5SGc3F1-PROC |
| **43** | 43.17 | 5.27 | 1693.1338^+2^ | -13.23 | 1693.1562^+2^ | H11N6F1-PROC |
| **44** | 44.13 | 2.1 | 1765.6500^+2^ | -14.10 | 1765.6749^+2^ | H11N6SAc1-PROC |
| **45** | 44.45 | 0.49 | 1838.1614^+2^ | -17.57 | 1838.1937^+2^ | H10N6SAc1SGc1-PROC *OR*  H9N6SGc2F1-PROC |
| **46** | 45.70 | 1.27 | 1875.6843^+2^ | -20.26 | 1875.7223^+2^ | H12N7F1-PROC |

Replicate 2

| **Peak #** | **Retention Time**  **R2** | **% Peak Areas** | **Observed M/Z Value**  **R2** | **Mass Error (PPM)** | **Calculated M/Z Value** | **Predicted Glycan Composition** |
| --- | --- | --- | --- | --- | --- | --- |
| **1** | 17.64 | 0.23 | 1479.6575^+1^ | 8.92 | 1479.6443^+1^ | H3N3F1-PROC |
| **2** | 19.89 | 0.5 | 841.8649^+2^ | -0.71 | 841.8655^+2^ | H3N4F1-PROC |
| **3** | 21.15 | 5.32 | 727.8089^+2^ | -1.51 | 727.81^+2^ | H5N2-PROC |
| **4** | 21.60 | 0.29 | 1454.6467^+1^ | 23.37 | 1454.6127^+1^ | H5N2-PROC |
|  |  |  | 943.4041^+2^ | -1.17 | 943.4052^+2^ | H3N5F1-PROC |
| **5** | 22.68 | 0.18 | 943.4066^+2^ | 1.48 | 943.4052^+2^ | H3N5F1-PROC |
| **6** | 23.14 | 0.35 | 829.3545^+2^ | 5.79 | 829.3497^+2^ | H5N3-PROC |
|  |  |  | 922.8953^+2^ | 3.68 | 922.8919^+2^ | H4N4F1-PROC |
| **7** | 23.63 | 0.26 | 922.8938^+2^ | 2.06 | 922.8919^+2^ | H4N4F1-PROC |
| **8** | 24.18 | 0.38 | 1044.9476^+2^ | 2.58 | 1044.9449^+2^ | H3N6F1-PROC |
| **9** | 24.71 | 4.47 | 808.8360^+2^ | -0.49 | 808.8364^+2^ | H6N2-PROC |
| **10** | 25.32 | 0.36 | 1024.4305^+2^ | -1.07 | 1024.4316^+2^ | H4N5F1-PROC |
| **11** | 26.37 | 0.32 | 1003.9083^+2^ | -9.96 | 1003.9183^+2^ | H5N4F1-PROC |
|  |  |  | 1068.4296^+2^ | -9.36 | 1068.4396^+2^ | H4N4SAc1F1-PROC |
| **12** | 26.65 | 0.41 | 1003.9195^+2^ | 1.20 | 1003.9183^+2^ | H5N4F1-PROC |
| **13** | 27.03 | 0.31 | 1003.9194^+2^ | 1.10 | 1003.9183^+2^ | H5N4F1-PROC |
| **14** | 28.14 | 5.41 | 889.8647^+2^ | 2.14 | 889.8628^+2^ | H7N2-PROC |
| **15** | 28.66 | 0.32 | 1105.4666^+2^ | 7.78 | 1105.458^+2^ | H5N5F1-PROC |
| **16** | 29.61 | 1.1 | 1084.9513^+2^ | 6.08 | 1084.9447^+2^ | H6N4F1-PROC |
| **17** | 30.80 | 1.65 | 1186.4916^+2^ | 6.07 | 1186.4844^+2^ | H6N5F1-PROC |
| **18** | 31.13 | 2.15 | 970.8895^+2^ | 0.31 | 970.8892^+2^ | H8N2-PROC |
|  |  |  | 1186.4852^+2^ | 0.67 | 1186.4844^+2^ | H6N5F1-PROC |
| **19** | 31.72 | 0.34 | 1157.4677^+2^ | 3.63 | 1157.4635^+2^ | H5N4SGc1F1-PROC |
| **20** | 32.49 | 7.21 | 1165.9777^+2^ | 5.66 | 1165.9711^+2^ | H7N4F1-PROC |
| **21** | 33.51 | 2.88 | 1051.9156^+2^ | 0.00 | 1051.9156^+2^ | H9N2-PROC |
|  |  |  | 1267.5184^+2^ | 6.00 | 1267.5108^+2^ | H7N5F1-PROC |
| **22** | 33.86 | 0.45 | 1267.5188^+2^ | 6.31 | 1267.5108^+2^ | H7N5F1-PROC |
| **23** | 34.22 | 2.16 | 1238.4973^+2^ | 5.98 | 1238.4899^+2^ | H6N4SGc1F1-PROC |
| **24** | 34.58 | 1 | 1340.5785^+2^ | 28.87 | 1340.5398^+2^ | H7N5F2-PROC |
|  |  |  | 1369.0565^+2^ | 4.38 | 1369.0505^+2^ | H7N6F1-PROC |
|  |  |  | 1404.5492^+2^ | -1.21 | 1404.5509^+2^ | H6N5SAc2-PROC |
| **25** | 35.00 | 1.59 | 1340.0387^+2^ | 6.79 | 1340.0296^+2^ | H6N5SGc1F1-PROC |
| **26** | 35.33 | 0.42 | 1340.0273^+2^ | -1.72 | 1340.0296^+2^ | H7N5SAc1-PROC *OR* H6N5SGc1F1-PROC |
| **27** | 35.89 | 5.76 | 1348.5438^+2^ | 4.89 | 1348.5372^+2^ | H8N5F1-PROC |
| **28** | 36.37 | 1.77 | 1311.0485^+2^ | 30.43 | 1311.0086^+2^ | H5N4SGc2F1-PROC |
|  |  |  | 1348.5415^+2^ | 3.19 | 1348.5372^+2^ | H8N5F1-PROC |
|  |  |  | 1421.0631^+2^ | 5.00 | 1421.056^+2^ | H8N5SAc1-PROC |
|  |  |  | 1550.0974^+2^ | -0.77 | 1550.0986^+2^ | H6N5SAc3-PROC |
| **29** | 36.77 | 1.36 | 1421.5702^+2^ | 2.81 | 1421.5662^+2^ | H8N5F2-PROC |
| **30** | 37.22 | 4.88 | 1421.0604^+2^ | 3.10 | 1421.056^+2^ | H8N5SAc1-PROC |
| **31** | 38.22 | 16.1 | 1429.5652^+2^ | 1.12 | 1429.5636^+2^ | H9N5F1-PROC |
| **32** | 38.57 | 2.87 | 1429.5664^+2^ | 1.96 | 1429.5636^+2^ | H9N5F1-PROC |
|  |  |  | 1502.0800^+2^ | -1.60 | 1502.0824^+2^ | H8N5SGc1F1-PROC |
| **33** | 39.09 | 0.68 | 1493.5742^+2^ | -0.33 | 1493.5747^+2^ | H7N5SAc1SGc1-PROC *OR* H6N5SGc2F1-PROC |
| **34** | 39.44 | 6.68 | 1502.0782^+2^ | -2.80 | 1502.0824^+2^ | H8N5SGc1F1-PROC |
| **35** | 40.03 | 1.7 | 1574.5947^+2^ | -4.13 | 1574.6012^+2^ | H7N5SGc2F1-PROC |
| **36** | 40.38 | 0.97 | 1574.5948^+2^ | -4.06 | 1574.6012^+2^ | H7N5SGc2F1-PROC |
|  |  |  | 1301.1641^+3^ | 0.61 | 1301.1633^+3^ | H7N6S4F1-PROC |
| **37** | *40.84* | *0.31* | *Not detected* | | | |
| **38** | 41.15 | 1.41 | 1574.5924^+2^ | -5.59 | 1574.6012^+2^ | H7N5SGc2F1-PROC |
| **39** | 41.30 | 3.21 | 1612.1207^+2^ | -5.58 | 1612.1297^+2^ | H10N6F1-PROC |
| **40** | 41.89 | 1.1 | 1647.1195^+2^ | -0.24 | 1647.1199^+2^ | H6N5SGc3F1-PROC |
| **41** | 42.32 | 1.16 | 1684.6416^+2^ | -4.10 | 1684.6485^+2^ | H10N6SAc1-PROC *OR*  H9N6SGcF1-PROC |
| **42** | 42.71 | 0.53 | 1647.1287^+2^ | 5.34 | 1647.1199^+2^ | H6N5SGc3F1-PROC |
| **43** | 43.16 | 5.78 | 1693.1350^+2^ | -12.52 | 1693.1562^+2^ | H11N6F1-PROC |
| **44** | 44.12 | 1.99 | 1765.6515^+2^ | -13.25 | 1765.6749^+2^ | H11N6SAc1-PROC |
| **45** | *44.45* | *0.5* | *Not detected* | | | |
| **46** | 45.68 | 1.17 | 1250.8219^+3^ | 3.68 | 1250.8173^+3^ | H12N7F1-PROC |

**Supplementary Table 2.** Comparison of Research Grade MOv18 IgE, technical Replicates 1 and 2. Shown are predicted monosaccharide compositions and suggested glycan structures based on observed MS m/z masses. H = hexose; N = N-acetylhexosamine; F = fucose; SGc = sialic acid (Neu5Gc); Sac = sialic acid (Neu5Ac).

**Supplementary Table 3**

Replicate 1

| **Peak #** | **R1**  **Retention Time (Min)** | **R1 % Peak Area** | **R1**  **Observed M/Z Value** | **Mass Error (PPM)** | **Calculated M/Z Value** | **Predicted Glycan Composition** |
| --- | --- | --- | --- | --- | --- | --- |
| **1** | 17.68 | 0.25 | 1479.6573^+1^ | 8.79 | 1479.6443^+1^ | H3N3F1-PROC |
| **2** | 19.95 | 0.55 | 841.8669^+2^ | 1.66 | 841.8655^+2^ | H3N4F1-PROC |
| **3** | 21.20 | 5.47 | 727.8098^+2^ | -0.27 | 727.81^+2^ | H5N2-PROC |
| **4** | 21.66 | 0.31 | 943.4081^+2^ | 3.07 | 943.4052^+2^ | H3N5F1-PROC |
| **5** | 22.72 | 0.22 | 943.4047^+2^ | -0.53 | 943.4052^+2^ | H3N5F1-PROC |
| **6** | 23.21 | 0.37 | 922.8964^+2^ | 4.88 | 922.8919^+2^ | H4N4F1-PROC |
| **7** | 23.70 | 0.28 | 922.8913^+2^ | -0.65 | 922.8919^+2^ | H4N4F1-PROC |
| **8** | 24.23 | 0.41 | 1044.9489^+2^ | 3.83 | 1044.9449^+2^ | H3N6F1-PROC |
| **9** | 24.76 | 4.56 | 808.8365^+2^ | 0.12 | 808.8364^+2^ | H6N2-PROC |
| **10** | 25.37 | 0.32 | 1024.4255^+2^ | -5.95 | 1024.4316^+2^ | H4N5F1-PROC |
| **11** | 26.46 | 0.27 | 1003.9204^+2^ | 2.09 | 1003.9183^+2^ | H5N4F1-PROC |
| **11** | 26.46 | 0.27 | 1068.4562^+2^ | 15.54 | 1068.4396^+2^ | H4N4SAc1F1-PROC |
| **12** | 26.71 | 0.42 | 1003.9197^+2^ | 1.39 | 1003.9183^+2^ | H5N4F1-PROC |
| **13** | 27.09 | 0.35 | 1003.9186^+2^ | 0.30 | 1003.9183^+2^ | H5N4F1-PROC |
| **14** | 28.20 | 5.51 | 889.8646^+2^ | 2.02 | 889.8628^+2^ | H7N2-PROC |
| **15** | 28.70 | 0.31 | 1105.4617^+2^ | 3.35 | 1105.458^+2^ | H5N5F1-PROC |
| **16** | 29.66 | 1.04 | 1084.9517^+2^ | 6.45 | 1084.9447^+2^ | H6N4F1-PROC |
| **17** | 30.85 | 1.6 | 1186.4911^+2^ | 5.65 | 1186.4844^+2^ | H6N5F1-PROC |
| **18** | 31.18 | 2.18 | 970.8893^+2^ | 0.10 | 970.8892^+2^ | H8N2-PROC |
| **18** | 31.18 | 2.18 | 1186.4875^+2^ | 2.61 | 1186.4844^+2^ | H6N5F1-PROC |
| **19** | 31.75 | 0.37 | 1157.4694^+2^ | 5.10 | 1157.4635^+2^ | H5N4SGc1F1-PROC |
| **20** | 32.54 | 7.19 | 1165.9779^+2^ | 5.83 | 1165.9711^+2^ | H7N4F1-PROC |
| **21** | 33.42 | 2.93 | 1267.5199^+2^ | 7.18 | 1267.5108^+2^ | H7N5F1-PROC |
| **22** | 33.91 | 0.35 | 1267.5233^+2^ | 9.86 | 1267.5108^+2^ | H7N5F1-PROC |
| **22** | 33.91 | 0.35 | 1332.0623^+2^ | 22.67 | 1332.0321^+2^ | H6N5S1F1-PROC |
| **23** | 34.26 | 2.17 | 1238.4985^+2^ | 6.94 | 1238.4899^+2^ | H6N4SGc1F1-PROC |
| **23** | 34.26 | 2.17 | 1340.0638^+2^ | 25.52 | 1340.0296^+2^ | H6N5SGc1F1-PROC |
| **24** | 34.65 | 1.11 | 1340.6038^+2^ | 47.74 | 1340.5398^+2^ | H7N5F2-PROC |
| **24** | 34.65 | 1.11 | 1369.0565^+2^ | 4.38 | 1369.0505^+2^ | H7N6F1-PROC |
| **24** | 34.65 | 1.11 | 1404.5526^+2^ | 1.21 | 1404.5509^+2^ | H6N5SAc2-PROC |
| **25** | 35.06 | 1.7 | 1340.0359^+2^ | 4.70 | 1340.0296^+2^ | H6N5SGc1F1-PROC |
| **26** | 35.38 | 0.45 | 1340.0687^+2^ | 29.18 | 1340.0296^+2^ | H6N5SGc1F1-PROC |
| **27** | 35.95 | 5.59 | 1348.5443^+2^ | 5.26 | 1348.5372^+2^ | H8N5F1-PROC |
| **28** | 36.41 | 1.75 | 1311.0103^+2^ | 1.30 | 1311.0086^+2^ | H5N4SGc2F1-PROC |
| **28** | 36.41 | 1.75 | 1348.5313^+2^ | -4.38 | 1348.5372^+2^ | H8N5F1-PROC |
| **28** | 36.41 | 1.75 | 1421.0569^+2^ | 0.63 | 1421.056^+2^ | H8N5SAc1-PROC |
| **28** | 36.41 | 1.75 | 1550.0956^+2^ | -1.94 | 1550.0986^+2^ | H6N5SAc3-PROC |
| **29** | 36.82 | 1.41 | 1421.5702^+2^ | 2.81 | 1421.5662^+2^ | H8N5F2-PROC |
| **30** | 37.29 | 4.8 | 1421.0616^+2^ | 3.94 | 1421.056^+2^ | H8N5SAc1-PROC |
| **31** | 38.28 | 15.94 | 1429.5656^+2^ | 1.40 | 1429.5636^+2^ | H9N5F1-PROC |
| **32** | 38.62 | 2.99 | 1429.5642^+2^ | 0.42 | 1429.5636^+2^ | H9N5F1-PROC |
| **32** | 38.62 | 2.99 | 1502.0807^+2^ | -1.13 | 1502.0824^+2^ | H8N5SGc1F1-PROC |
| **32** | 38.62 | 2.99 | 1510.5930^+2^ | 1.92 | 1510.5901^+2^ | H10N5F1 |
| **33** | 39.13 | 0.98 | 1493.5732^+2^ | -1.00 | 1493.5747^+2^ | H6N5SGc2F1-PROC |
| **34** | 39.48 | 6.98 | 1502.0792^+2^ | -2.13 | 1502.0824^+2^ | H8N5SGc1F1-PROC |
| **35** | 40.07 | 1.77 | 1574.5934^+2^ | -4.95 | 1574.6012^+2^ | H7N5SGc2F1-PROC |
| **36** | 40.37 | 0.98 | 1574.6067^+2^ | 3.49 | 1574.6012^+2^ | H7N5SGc2F1-PROC |
| **36** | 40.37 | 0.98 | 1301.1949^+3^ | 24.29 | 1301.1633^+3^ | H7N6SAc4F1-PROC |
| ***37*** | *40.87* | *0.35* | *Not detected* | | | |
| **38** | 41.18 | 1.83 | 1574.5933^+2^ | -5.02 | 1574.6012^+2^ | H7N5SGc2F1-PROC |
| **39** | 41.36 | 2.73 | 1574.5797^+2^ | -13.65 | 1574.6012^+2^ | H7N5SGc2F1-PROC |
| **39** | 41.36 | 2.73 | 1612.1221^+2^ | -4.71 | 1612.1297^+2^ | H10N6F1-PROC |
| **39** | 41.36 | 2.73 | 1623.1171^+2^ | -6.41 | 1623.1275^+2^ | H6N5SAc3F1-PROC |
| **40** | 41.93 | 1.09 | 1647.1157^+2^ | -2.55 | 1647.1199^+2^ | H6N5SGc3F1-PROC |
| **40** | 41.93 | 1.09 | 1685.1830^+2^ | 14.42 | 1685.1587^+2^ | H10N6F2-PROC |
| **41** | 42.37 | 1.09 | 1684.6505^+2^ | 1.19 | 1684.6485^+2^ | H10N6SAc1-PROC *OR*  H9N6SGcF1-PROC |
| **42** | 42.73 | 0.41 | 1647.6108^+2^ | -11.71 | 1647.6301^+2^ | H7N5SGc2F2-PROC |
| **43** | 43.22 | 5.4 | 1693.1334^+2^ | -13.47 | 1693.1562^+2^ | H11N6F1-PROC |
| **44** | 44.18 | 1.84 | 1765.6515^+2^ | -13.25 | 1765.6749^+2^ | H11N6SAc1-PROC |
| **45** | 44.48 | 0.41 | 1838.1693^+2^ | -13.27 | 1838.1937^+2^ | H10N6SAc1SGc1-PROC *OR*  H9N6SGc2F1-PROC |
| **46** | *45.76* | *0.96* | *Not detected* | | | |

Replicate 2

| **Peak #** | **R2**  **Retention Time (Min)** | **R2 % Peak Area** | **R2**  **Observed M/Z Value** | **Mass Error (PPM)** | **Calculated M/Z Value** | **Predicted Glycan Composition** |
| --- | --- | --- | --- | --- | --- | --- |
| **1** | 17.69 | 0.28 | 1682.7686^+1^ | 26.68 | 1682.7237^+1^ | H3N4F1-PROC |
| **2** | 19.94 | 0.55 | 841.8668^+2^ | 1.54 | 841.8655^+2^ | H3N4F1-PROC |
| **3** | 21.20 | 5.54 | 727.8100^+2^ | 0.00 | 727.81^+2^ | H5N2-PROC |
| **4** | 21.67 | 0.37 | 943.4059^+2^ | 0.74 | 943.4052^+2^ | H3N5F1-PROC |
| **5** | 22.72 | 0.3 | 943.9023^+2^ | 526.92 | 943.4052^+2^ | H3N5F1-PROC |
| **6** | 23.17 | 0.47 | 829.3568^+2^ | 8.56 | 829.3497^+2^ | H5N3-PROC |
| **6** | 23.17 | 0.47 | 922.8929^+2^ | 1.08 | 922.8919^+2^ | H4N4F1-PROC |
| **7** | 23.69 | 0.32 | 922.8956^+2^ | 4.01 | 922.8919^+2^ | H4N4F1-PROC |
| **8** | 24.23 | 0.42 | 1044.9496^+2^ | 4.50 | 1044.9449^+2^ | H3N6F1-PROC |
| **9** | 24.76 | 4.69 | 808.8375^+2^ | 1.36 | 808.8364^+2^ | H6N2-PROC |
| **10** | 25.34 | 0.34 | 1024.4153^+2^ | -15.91 | 1024.4316^+2^ | H4N5F1-PROC |
| **11** | 26.45 | 0.32 | 1003.9316^+2^ | 13.25 | 1003.9183^+2^ | H5N4F1-PROC |
| **12** | 26.71 | 0.51 | 1003.9179^+2^ | -0.40 | 1003.9183^+2^ | H5N4F1-PROC |
| **13** | 27.07 | 0.34 | 1003.9166^+2^ | -1.69 | 1003.9183^+2^ | H5N4F1-PROC |
| **14** | 28.19 | 5.6 | 889.8634^+2^ | 0.67 | 889.8628^+2^ | H7N2-PROC |
| **15** | 28.72 | 0.39 | 1105.4572^+2^ | -0.72 | 1105.458^+2^ | H5N5F1-PROC |
| **16** | 29.67 | 1.46 | 1084.9503^+2^ | 5.16 | 1084.9447^+2^ | H6N4F1-PROC |
| **17** | 30.85 | 1.59 | 1186.4912^+2^ | 5.73 | 1186.4844^+2^ | H6N5F1-PROC |
| **18** | 31.20 | 2.26 | 970.8896^+2^ | 0.41 | 970.8892^+2^ | H8N2-PROC |
| **18** | 31.20 | 2.26 | 1186.4802^+2^ | -3.54 | 1186.4844^+2^ | H6N5F1-PROC |
| **19** | 31.74 | 0.34 | 1157.4668^+2^ | 2.85 | 1157.4635^+2^ | H5N4SGc1F1-PROC |
| **20** | 32.54 | 6.93 | 1165.9783^+2^ | 6.18 | 1165.9711^+2^ | H7N4F1-PROC |
| **21** | 33.57 | 3.12 | 1267.5195^+2^ | 6.86 | 1267.5108^+2^ | H7N5F1-PROC |
| **22** | 33.94 | 0.3 | 1267.5164^+2^ | 4.42 | 1267.5108^+2^ | H7N5F1-PROC |
| **23** | 34.27 | 2.32 | 1238.4993^+2^ | 7.59 | 1238.4899^+2^ | H6N4SGc1F1-PROC |
| **24** | 34.64 | 1.09 | 1340.5488^+2^ | 6.71 | 1340.5398^+2^ | H7N5F2-PROC |
| **24** | 34.64 | 1.09 | 1369.0559^+2^ | 3.94 | 1369.0505^+2^ | H7N6F1-PROC |
| **24** | 34.64 | 1.09 | 1404.5493^+2^ | -1.14 | 1404.5509^+2^ | H6N5SAc2-PROC |
| **25** | 35.05 | 1.81 | 1340.0371^+2^ | 5.60 | 1340.0296^+2^ | H6N5SGc1F1-PROC |
| **26** | 35.34 | 0.41 | 1340.0697^+2^ | 29.92 | 1340.0296^+2^ | H6N5SGc1F1-PROC |
| **27** | 35.96 | 5.84 | 1348.5446^+2^ | 5.49 | 1348.5372^+2^ | H8N5F1-PROC |
| **28** | 36.40 | 1.9 | 1348.5907^+2^ | 39.67 | 1348.5372^+2^ | H8N5F1-PROC |
| **28** | 36.40 | 1.9 | 1421.0563^+2^ | 0.21 | 1421.056^+2^ | H8N5SAc1-PROC |
| **28** | 36.40 | 1.9 | 1550.1021^+2^ | 2.26 | 1550.0986^+2^ | H6N5SAc3-PROC |
| **29** | 36.82 | 1.39 | 1421.5707^+2^ | 3.17 | 1421.5662^+2^ | H8N5F2-PROC |
| **30** | 37.28 | 5.02 | 1421.0616^+2^ | 3.94 | 1421.056^+2^ | H8N5SAc1-PROC |
| **31** | 38.28 | 15.42 | 1429.5648^+2^ | 0.84 | 1429.5636^+2^ | H9N5F1-PROC |
| **32** | 38.62 | 2.88 | 1429.5667^+2^ | 2.17 | 1429.5636^+2^ | H9N5F1-PROC |
| **32** | 38.62 | 2.88 | 1502.0802^+2^ | -1.46 | 1502.0824^+2^ | H8N5SGc1F1-PROC |
| **33** | 39.15 | 0.72 | 1493.5709^+2^ | -2.54 | 1493.5747^+2^ | H6N5SGc2F1-PROC |
| **34** | 39.48 | 7.04 | 1502.0797^+2^ | -1.80 | 1502.0824^+2^ | H8N5SGc1F1-PROC |
| **35** | 40.07 | 1.76 | 1574.5942^+2^ | -4.45 | 1574.6012^+2^ | H7N5SGc2F1-PROC |
| **36** | 40.38 | 0.99 | 1574.6283^+2^ | 17.21 | 1574.6012^+2^ | H7N5SGc2F1-PROC |
| ***37*** | *40.88* | *0.31* | *Not detected* | | | |
| **38** | 41.20 | 1.55 | 1574.5953^+2^ | -3.75 | 1574.6012^+2^ | H7N5SGc2F1-PROC |
| **39** | 41.36 | 2.48 | 1574.6003^+2^ | -0.57 | 1574.6012^+2^ | H7N5SGc2F1-PROC |
| **39** | 41.36 | 2.48 | 1612.1281^+2^ | -0.99 | 1612.1297^+2^ | H10N6F1-PROC |
| **40** | 41.93 | 1.07 | 1647.1183^+2^ | -0.97 | 1647.1199^+2^ | H6N5SGc3F1-PROC |
| **41** | 42.35 | 1.08 | 1684.6272^+2^ | -12.64 | 1684.6485^+2^ | H10N6SAc1-PROC *OR*  H9N6SGcF1-PROC |
| **42** | *42.74* | *0.63* | *Not detected* | | | |
| **43** | 43.22 | 4.87 | 1693.1362^+2^ | -11.81 | 1693.1562^+2^ | H11N6F1-PROC |
| **44** | 44.18 | 1.7 | 1765.6499^+2^ | -14.16 | 1765.6749^+2^ | H11N6SAc1-PROC |
| ***45*** | *44.51* | *0.43* | *Not detected* | | | |
| ***46*** | *45.76* | *0.84* | *Not detected* | | | |

**Supplementary Table 3.** Predicted monosaccharide compositions and suggested glycan structures for Research Grade MOv18 IgE - Untreated, technical Replicates 1 and 2 based on observed MS m/z masses. H = hexose; N = N-acetylhexosamine; F = fucose; SGc = sialic acid (Neu5Gc); SAc = sialic acid (Neu5Ac).

**Supplementary Table 4**

Replicate 1

| **Peak #** | **R1**  **Retention Time (Min)** | **R1 % Peak Area** | **R1**  **Observed M/Z Value** | **Mass Error (PPM)** | **Calculated M/Z Value** | **Predicted Glycan Composition** |
| --- | --- | --- | --- | --- | --- | --- |
| **1** | 17.69 | 0.24 | 1479.6549^+1^ | 7.16 | 1479.6443^+1^ | H3N3F1-PROC |
| **2** | 19.95 | 0.53 | 841.8677^+2^ | 2.61 | 841.8655^+2^ | H3N4F1-PROC |
| **3** | 21.20 | 5.49 | 727.8096^+2^ | -0.55 | 727.81^+2^ | H5N2-PROC |
| **4** | 21.65 | 0.39 | 943.4053^+2^ | 0.11 | 943.4052^+2^ | H3N5F1-PROC |
| **5** | 22.73 | 0.28 | 943.4067^+2^ | 1.59 | 943.4052^+2^ | H3N5F1-PROC |
| **6** | 23.20 | 0.52 | 829.3508^+2^ | 1.33 | 829.3497^+2^ | H5N3-PROC |
|  |  |  | 922.8945^+2^ | 2.82 | 922.8919^+2^ | H4N4F1-PROC |
| **7** | 23.69 | 0.53 | 922.8943^+2^ | 2.60 | 922.8919^+2^ | H4N4F1-PROC |
| **8** | 24.23 | 0.41 | 1044.9493^+2^ | 4.21 | 1044.9449^+2^ | H3N6F1-PROC |
| **9** | 24.75 | 4.62 | 808.8366^+2^ | 0.25 | 808.8364^+2^ | H6N2-PROC |
| **10** | 25.38 | 0.98 | 1024.4335^+2^ | 1.85 | 1024.4316^+2^ | H4N5F1-PROC |
| **11** | 26.71 | 7.16 | 1003.9171^+2^ | -1.20 | 1003.9183^+2^ | H5N4F1-PROC |
| **12** | 28.20 | 5.71 | 889.8647^+2^ | 2.14 | 889.8628^+2^ | H7N2-PROC |
| **13** | 29.49 | 1.02 | 1207.0051^+2^ | 6.13 | 1206.9977^+2^ | H5N6F1-PROC |
| **14** | 30.31 | 0.89 | 1149.4639^+2^ | -1.83 | 1149.466^+2^ | H5N4SAc1F1-PROC |
|  |  |  | 1177.9801^+2^ | 2.80 | 1177.9768^+2^ | H4N5SGc1F1-PROC |
| **15** | 30.86 | 22.35 | 1186.4895^+2^ | 4.30 | 1186.4844^+2^ | H6N5F1-PROC |
| **16** | 31.17 | 2.02 | 970.8863^+2^ | -2.99 | 970.8892^+2^ | H8N2-PROC |
|  |  |  | 1186.4912^+2^ | 5.73 | 1186.4844^+2^ | H6N5F1-PROC |
| **17** | 31.75 | 1.87 | 1157.4729^+2^ | 8.12 | 1157.4635^+2^ | H5N4SGc1F1-PROC |
| **18** | 31.94 | 1.65 | 1157.4631^+2^ | -0.35 | 1157.4635^+2^ | H5N4SGc1F1-PROC |
|  |  |  | 1259.5071^+2^ | -5.00 | 1259.5134^+2^ | H6N5F2-PROC |
| **19** | 32.56 | 0.91 | 1259.0065^+2^ | 2.62 | 1259.0032^+2^ | H6N5S1-PROC |
|  |  |  | 1332.0836^+2^ | 38.66 | 1332.0321^+2^ | H6N5SAc1F1-PROC |
| **20** | 32.82 | 0.52 | 1259.0089^+2^ | 4.53 | 1259.0032^+2^ | H6N5S1-PROC |
|  |  |  | 1287.5959^+2^ | 63.69 | 1287.5139^+2^ | H5N6SGc1-PROC |
|  |  |  | 1332.0427^+2^ | 7.96 | 1332.0321^+2^ | H6N5SAc1F1-PROC |
|  |  |  | 1360.5417^+2^ | -0.88 | 1360.5429^+2^ | H6N6SAc1-PROC OR  H5N6SGc1-F1-PROC |
| **21** | 33.38 | 0.46 | 1051.9178^+2^ | 2.09 | 1051.9156^+2^ | H9N2-PROC |
| **22** | 33.80 | 1.71 | 1332.0402^+2^ | 6.08 | 1332.0321^+2^ | H6N5SAc1F1-PROC |
| **23** | 34.18 | 3.20 | 1340.0345^+2^ | 3.66 | 1340.0296^+2^ | H6N5SGc1F1-PROC |
| **24** | 34.58 | 0.53 | 1340.0818^+2^ | 38.95 | 1340.0296^+2^ | H7N5SAc1-PROC *OR* H6N5SGc1F1-PROC |
|  |  |  | 1404.5564^+2^ | 3.92 | 1404.5509^+2^ | H6N5SAc2-PROC |
| **25** | 35.03 | 19.00 | 1369.0552^+2^ | 3.43 | 1369.0505^+2^ | H7N6F1-PROC |
| **26** | 35.83 | 1.54 | 1404.5432^+2^ | -5.48 | 1404.5509^+2^ | H6N5SAc2-PROC |
|  |  |  | 1413.0668^+2^ | 5.87 | 1413.0585^+2^ | H6N5SGc1F2-PROC |
|  |  |  | 1442.0842^+2^ | 3.26 | 1442.0795^+2^ | H7N6F2-PROC |
| **27** | 36.37 | 1.04 | 1311.0377^+2^ | 22.20 | 1311.0086^+2^ | H5N4SGc2F1-PROC *OR* H6N4SAc1SGc1-PROC |
|  |  |  | 1550.0911^+2^ | -4.84 | 1550.0986^+2^ | H6N5SAc3-PROC |
| **28** | 36.70 | 0.37 | 1485.5856^+2^ | 5.59 | 1485.5773^+2^ | H7N5S2-PROC OR H5N5S2F2-PROC |
| **29** | 37.00 | 0.30 | 1485.5488^+2^ | -19.18 | 1485.5773^+2^ | H7N5SAc2-PROC OR H5N5SGc2F2-PROC |
| **30** | 37.35 | 0.73 | 1493.5673^+2^ | -4.95 | 1493.5747^+2^ | H7N5SAc1SGc1-PROC *OR* H6N5SGc2F1-PROC |
|  |  |  | 1514.5854^+2^ | -8.45 | 1514.5982^+2^ | H7N6SAc1F1-PROC |
| **31** | 37.65 | 1.92 | 1522.5850^+2^ | -7.03 | 1522.5957^+2^ | H7N6SGc1F1-PROC |
|  |  |  | 1550.0969^+2^ | -1.10 | 1550.0986^+2^ | H6N5SAc3-PROC |
| **32** | 38.14 | 2.04 | 1493.5712^+2^ | -2.34 | 1493.5747^+2^ | H7N5SAc1SGc1-PROC *OR* H6N5SGc2F1-PROC |
| **33** | 38.51 | 4.09 | 1522.5885^+2^ | -4.73 | 1522.5957^+2^ | H7N6SGc1F1-PROC |
|  |  |  | 1034.7550^+3^ | 7.92 | 1034.7468^+3^ | H8N7F1-PROC |
| **34** | 39.12 | 3.01 | 1493.5715^+2^ | -2.14 | 1493.5747^+2^ | H6N5SGc2F1-PROC |
| **35** | 41.15 | 0.96 | 1676.1304^+2^ | -6.20 | 1676.1408^+2^ | H8N6SAc1SGc1-PROC *OR* H7N6SGc2F1-PROC |
| **36** | 41.47 | 0.37 | 1137.1028^+3^ | -6.60 | 1137.1103^+3^ | H9N7SAc1-PROC OR H8N7SGc1F1-PROC |
| **37** | 41.91 | 0.65 | 1647.1333^+2^ | 8.14 | 1647.1199^+2^ | H6N5SGc3F1-PROC |

Replicate 2

| **Peak #** | **R2**  **Retention Time (Min)** | **R2 % Peak Area** | **R2**  **Observed M/Z Value** | **Mass Error (PPM)** | **Calculated M/Z Value** | **Predicted Glycan Composition** |
| --- | --- | --- | --- | --- | --- | --- |
| **1** | 17.67 | 0.30 | 1479.6555^+1^ | 7.57 | 1479.6443^+1^ | H3N3F1-PROC |
| **2** | 19.97 | 0.55 | 841.8677^+2^ | 2.61 | 841.8655^+2^ | H3N4F1-PROC |
| **3** | 21.20 | 5.28 | 727.8103^+2^ | 0.41 | 727.81^+2^ | H5N2-PROC |
| **4** | 21.66 | 0.40 | 943.4103^+2^ | 5.41 | 943.4052^+2^ | H3N5F1-PROC |
| **5** | 22.73 | 0.28 | 943.3950^+2^ | -10.81 | 943.4052^+2^ | H3N5F1-PROC |
| **6** | 23.21 | 0.56 | 829.3518^+2^ | 2.53 | 829.3497^+2^ | H5N3-PROC |
|  |  |  | 922.8909^+2^ | -1.08 | 922.8919^+2^ | H4N4F1-PROC |
| **7** | 23.70 | 0.50 | 922.8962^+2^ | 4.66 | 922.8919^+2^ | H4N4F1-PROC |
| **8** | 24.23 | 0.43 | 1044.9475^+2^ | 2.49 | 1044.9449^+2^ | H3N6F1-PROC |
| **9** | 24.77 | 4.54 | 808.8377^+2^ | 1.61 | 808.8364^+2^ | H6N2-PROC |
| **10** | 25.38 | 1.08 | 1024.4337^+2^ | 2.05 | 1024.4316^+2^ | H4N5F1-PROC |
| **11** | 26.71 | 7.00 | 1003.9178^+2^ | -0.50 | 1003.9183^+2^ | H5N4F1-PROC |
| **12** | 28.19 | 5.68 | 889.8666^+2^ | 4.27 | 889.8628^+2^ | H7N2-PROC |
| **13** | 29.52 | 1.02 | 1207.0056^+2^ | 6.55 | 1206.9977^+2^ | H5N6F1-PROC |
| **14** | 30.27 | 0.91 | 1149.4547^+2^ | -9.83 | 1149.466^+2^ | H5N4S1F1-PROC |
|  |  |  | 1177.9713^+2^ | -4.67 | 1177.9768^+2^ | H5N5SAc1-PROC  *OR*  H4N5SgC1F1-PROC |
| **15** | 30.86 | 22.07 | 1186.4900^+2^ | 4.72 | 1186.4844^+2^ | H6N5F1-PROC |
| **16** | 31.17 | 2.20 | 970.8916^+2^ | 2.47 | 970.8892^+2^ | H8N2-PROC |
|  |  |  | 1186.4932^+2^ | 7.42 | 1186.4844^+2^ | H6N5F1-PROC |
| **17** | 31.76 | 2.04 | 1157.4734^+2^ | 8.55 | 1157.4635^+2^ | H5N4SGc1F1-PROC |
| **18** | 31.95 | 1.64 | 1259.5227^+2^ | 412.63 | 1259.0032^+2^ | H6N5S1-PROC |
| **19** | 32.58 | 0.86 | 1259.0093^+2^ | 4.85 | 1259.0032^+2^ | H6N5S1-PROC |
|  |  |  | 1332.0403^+2^ | 6.16 | 1332.0321^+2^ | H6N5SAc1F1-PROC |
| **20** | 32.82 | 0.62 | 1258.9984^+2^ | -3.81 | 1259.0032^+2^ | H6N5S1-PROC |
|  |  |  | 1288.0349^+2^ | 8.38 | 1288.0241^+2^ | H6N6F1-PROC |
|  |  |  | 1360.5432^+2^ | 0.22 | 1360.5429^+2^ | H6N6SAc1-PROC  *OR* H5N6SGc1-F1-PROC |
| **21** | 33.39 | 0.56 | 1051.9287^+2^ | 12.45 | 1051.9156^+2^ | H9N2-PROC |
| **22** | 33.80 | 1.71 | 1332.0408^+2^ | 6.53 | 1332.0321^+2^ | H6N5SAc1F1-PROC |
| **23** | 34.18 | 3.17 | 1340.0355^+2^ | 4.40 | 1340.0296^+2^ | H6N5SGc1F1-PROC |
| **24** | 34.58 | 0.68 | 1340.0829^+2^ | 39.78 | 1340.0296^+2^ | H7N5SAc1-PROC *OR* H6N5SGc1F1-PROC |
|  |  |  | 1404.5554^+2^ | 3.20 | 1404.5509^+2^ | H6N5SAc2-PROC |
| **25** | 35.03 | 18.66 | 1369.0562^+2^ | 4.16 | 1369.0505^+2^ | H7N6F1-PROC |
| **26** | 35.83 | 1.69 | 1404.5416^+2^ | -6.62 | 1404.5509^+2^ | H6N5SAc2-PROC |
|  |  |  | 1412.5469^+2^ | -0.99 | 1412.5483^+2^ | H5N5SGc2F1-PROC |
|  |  |  | 1442.0734^+2^ | -4.23 | 1442.0795^+2^ | H7N6F2-PROC |
| **27** | 36.39 | 1.11 | 1311.0583^+2^ | 37.91 | 1311.0086^+2^ | H5N4SGc2F1-PROC  *OR* H6N4SAc1SGc1-PROC |
|  |  |  | 1550.0942^+2^ | -2.84 | 1550.0986^+2^ | H6N5SAc3-PROC |
| **28** | 36.71 | 0.42 | 1485.5554^+2^ | -14.74 | 1485.5773^+2^ | H7N5S2-PROC *OR* H5N5S2F2-PROC |
| **29** | 37.00 | 0.30 | 1485.5787^+2^ | 0.94 | 1485.5773^+2^ | H7N5SAc2-PROC  *OR* H5N5SGc2F2-PROC |
| **30** | 37.35 | 0.80 | 1493.5640^+2^ | -7.16 | 1493.5747^+2^ | H7N5SAc1SGc1-PROC  *OR* H6N5SGc2F1-PROC |
|  |  |  | 1514.5842^+2^ | -9.24 | 1514.5982^+2^ | H7N6SAc1F1-PROC |
| **31** | 37.65 | 1.89 | 1522.5865^+2^ | -6.04 | 1522.5957^+2^ | H7N6SGc1F1-PROC |
|  |  |  | 1550.0941^+2^ | -2.90 | 1550.0986^+2^ | H6N5SAc3-PROC |
| **32** | 38.15 | 2.09 | 1493.5715^+2^ | -2.14 | 1493.5747^+2^ | H6N5SGc2F1-PROC |
| **33** | 38.51 | 4.17 | 1522.5885^+2^ | -4.73 | 1522.5957^+2^ | H7N6SGc1F1-PROC |
|  |  |  | 1034.7563^+3^ | 9.18 | 1034.7468^+3^ | H8N7F1-PROC |
| **34** | 39.11 | 2.83 | 1493.5734^+2^ | -0.87 | 1493.5747^+2^ | H6N5SGc2F1-PROC |
| **35** | 41.13 | 0.95 | 1676.1569^+2^ | 9.61 | 1676.1408^+2^ | H8N6SAc1SGc1-PROC  *OR* H7N6SGc2F1-PROC |
| **36** | 41.48 | 0.36 | 1137.4601^+3^ | 307.62 | 1137.1103^+3^ | H9N7SAc1-PROC  *OR* H8N7SGc1F1-PROC |
| **37** | 41.91 | 0.66 | 1647.0980^+2^ | -13.30 | 1647.1199^+2^ | H6N5SGc3F1-PROC |

**Supplementary Table 4.** Predicted monosaccharide compositions and suggested glycan structures for Research Grade Alpha Galactosidase Treated MOv18 IgE technical replicates 1 and 2 based on observed MS m/z masses. H = hexose; N = N-acetylhexosamine; F = fucose; SGc = sialic acid (Neu5Gc); Sac = sialic acid (Neu5Ac).

**Supplementary Table 5**

| **Research Grade MOv18 IgE - Untreated** | | | | **Research Grade MOv18 IgE - Alpha Galactosidase Treated** | | | | **Calculated M/Z Value** |
| --- | --- | --- | --- | --- | --- | --- | --- | --- |
| **Peak #** | **Retention Time (Min)** | **% Peak Area** | **Observed M/Z Value** | **Peak #** | **Retention Time (Min)** | **% Peak Area** | **Observed M/Z Value** |  |
| **1** | 17.68 | 0.25 | 1479.6573^+1^ | **1** | 17.69 | 0.24 | 1479.6549^+1^ | 1479.6443^+1^ H3N3F1-PROC |
| **2** | 19.95 | 0.55 | 841.8669^+2^ | **2** | 19.95 | 0.53 | 841.8677^+2^ | 841.8655^+2^ H3N4F1-PROC |
| **3** | 21.20 | 5.47 | 727.8098^+2^ | **3** | 21.20 | 5.49 | 727.8096^+2^ | 727.81^+2^ H5N2-PROC |
| **4** | 21.66 | 0.31 | 943.4081^+2^ | **4** | 21.65 | 0.39 | 943.4053^+2^ | 943.4052^+2^ H3N5F1-PROC |
| **5** | 22.72 | 0.22 | 943.4047^+2^ | **5** | 22.73 | 0.28 | 943.4067^+2^ | 943.4052^+2^ H3N5F1-PROC |
|  | | | | **6** | 23.20 | 0.52 | 829.3508^+2^ | 829.3497^+2^ H5N3-PROC |
| **6** | 23.21 | 0.37 | 922.8964^+2^ |  |  |  | 922.8945^+2^ | 922.8919^+2^ H4N4F1-PROC |
| **7** | 23.70 | 0.28 | 922.8913^+2^ | **7** | 23.69 | 0.53 | 922.8943^+2^ | 922.8919^+2^ H4N4F1-PROC |
| **8** | 24.23 | 0.41 | 1044.9489^+2^ | **8** | 24.23 | 0.41 | 1044.9493^+2^ | 1044.9449^+2^ H3N6F1-PROC |
| **9** | 24.76 | 4.56 | 808.8365^+2^ | **9** | 24.75 | 4.62 | 808.8366^+2^ | 808.8364^+2^ H6N2-PROC |
| **10** | 25.37 | 0.32 | 1024.4255^+2^ | **10** | 25.38 | 0.98 | 1024.4335^+2^ | 1024.4316^+2^ H4N5F1-PROC |
| **11** | 26.46 | 0.27 | 1003.9204^+2^ |  | | | | 1003.9183^+2^ H5N4F1-PROC |
| **11** | 26.46 | 0.27 | 1068.4562^+2^ |  |  |  |  | 1068.4396^+2^ H4N4SAc1F1-PROC |
|  | | | | **11** | 26.71 | 7.16 | 1003.9171^+2^ | 1003.9183^+2^ H5N4F1-PROC |
| **12** | 26.71 | 0.42 | 1003.9197^+2^ |  | | | | 1003.9183^+2^ H5N4F1-PROC |
| **13** | 27.09 | 0.35 | 1003.9186^+2^ |  |  |  |  | 1003.9183^+2^ H5N4F1-PROC |
| **14** | 28.20 | 5.51 | 889.8646^+2^ | **12** | 28.20 | 5.71 | 889.8647^+2^ | 889.8628^+2^ H7N2-PROC |
| **15** | 28.70 | 0.31 | 1105.4617^+2^ |  | | | | 1105.458^+2^ H5N5F1-PROC |
|  | | | | **13** | 29.49 | 1.02 | 1207.0051^+2^ | 1206.9977^+2^ H5N6F1-PROC |
| **16** | 29.66 | 1.04 | 1084.9517^+2^ |  | | | | 1084.9447^+2^ H6N4F1-PROC |
|  | | | | **14** | 30.31 | 0.89 | 1149.4639^+2^ | 1149.466^+2^ H5N4SAc1F1-PROC |
|  |  |  |  |  |  |  | 1177.9801^+2^ | 1177.9768^+2^ H4N5SGc1F1-PROC |
| **17** | 30.85 | 1.6 | 1186.4911^+2^ | **15** | 30.86 | 22.35 | 1186.4895^+2^ | 1186.4844^+2^ H6N5F1-PROC |
| **18** | 31.18 | 2.18 | 970.8893^+2^ | **16** | 31.17 | 2.02 | 970.8863^+2^ | 970.8892^+2^ H8N2-PROC |
|  |  |  | 1186.4875^+2^ |  |  |  | 1186.4912^+2^ | 1186.4844^+2^ H6N5F1-PROC |
| **19** | 31.75 | 0.37 | 1157.4694^+2^ | **17** | 31.75 | 1.87 | 1157.4729^+2^ | 1157.4635^+2^ H5N4SGc1F1-PROC |
|  | | | | **18** | 31.94 | 1.65 | 1157.4631^+2^ | 1157.4635^+2^ H5N4SGc1F1-PROC |
|  |  |  |  |  |  |  | 1259.5071^+2^ | 1259.5134 H6N5F2-PROC |
| **20** | 32.54 | 7.19 | 1165.9779^+2^ | **19** |  | | | 1165.9711^+2^ H7N4F1-PROC |
|  | | | |  | 32.56 | 0.91 | 1259.0065^+2^ | 1259.0032^+2^ H6N5S1-PROC |
|  |  |  |  |  |  |  | 1332.0836^+2^ | 1332.0321^+2^ H6N5SAc1F1-PROC |
|  |  |  |  | **20** | 32.82 | 0.52 | 1259.0089^+2^ | 1259.0032^+2^ H6N5S1-PROC |
|  |  |  |  |  |  |  | 1287.5959^+2^ | 1287.5139^+2^ H5N6SGc1-PROC |
|  |  |  |  |  |  |  | 1332.0427^+2^ | 1332.0321^+2^ H6N5SAc1F1-PROC |
|  |  |  |  |  |  |  | 1360.5417^+2^ | 1360.5429^+2^ H6N6SAc1-PROC *OR*  H5N6SGc1-F1-PROC |
|  |  |  |  | **21** | 33.38 | 0.46 | 1051.9178^+2^ | 1051.9156^+2^ H9N2-PROC |
| **21** | 33.42 | 2.93 | 1267.5199^+2^ |  | | | | 1267.5108^+2^ H7N5F1-PROC |
| **22** | 33.91 | 0.35 | 1267.5233^+2^ |  |  |  |  | 1267.5108^+2^ H7N5F1-PROC |
|  |  |  | 1332.0623^+2^ | **22** | 33.80 | 1.71 | 1332.0402^+2^ | 1332.0321^+2^ H6N5S1F1-PROC |
| **23** | 34.26 | 2.17 | 1238.4985^+2^ |  | | | | 1238.4899^+2^ H6N4SGc1F1-PROC |
|  |  |  | 1340.0638^+2^ | **23** | 34.18 | 3.20 | 1340.0345^+2^ | 1340.0296^+2^ H6N5SGc1F1-PROC |
| **24** | 34.65 | 1.11 | 1340.6038^+2^ | **24** | 34.58 | 0.53 | 1340.0818^+2^ | 1340.5398^+2^ H7N5F2-PROC |
|  |  |  | 1369.0565^+2^ |  |  | | | 1369.0505^+2^ H7N6F1-PROC |
|  |  |  | 1404.5526^+2^ |  | 34.58 | 0.53 | 1404.5564^+2^ | 1404.5509^+2^ H6N5SAc2-PROC |
| **25** | 35.06 | 1.7 | 1340.0359^+2^ |  | | | | 1340.0296^+2^ H6N5SGc1F1-PROC |
|  | | | | **25** | 35.03 | 19.00 | 1369.0552^+2^ | 1369.0505^+2^ H7N6F1-PROC |
| **26** | 35.38 | 0.45 | 1340.0687^+2^ |  | | | | 1340.0296^+2^ H6N5SGc1F1-PROC |
|  | | | | **26** | 35.83 | 1.54 | 1404.5432^+2^ | 1404.5509^+2^ H6N5SAc2-PROC |
|  |  |  |  |  |  |  | 1413.0668^+2^ | 1413.0585^+2^ H6N5SGc1F2-PROC |
|  |  |  |  |  |  |  | 1442.0842^+2^ | 1442.0795^+2^ H7N6F2-PROC |
| **27** | 35.95 | 5.59 | 1348.5443^+2^ |  | | | | 1348.5372^+2^ H8N5F1-PROC |
| **28** | 36.41 | 1.75 | 1311.0103^+2^ | **27** | 36.37 | 1.04 | 1311.0377^+2^ | 1311.0086^+2^ H5N4SGc2F1-PROC |
|  |  |  | 1348.5313^+2^ |  |  | | | 1348.5372^+2^ H8N5F1-PROC |
|  |  |  | 1421.0569^+2^ |  |  |  |  | 1421.056^+2^ H8N5SAc1-PROC |
|  |  |  | 1550.0956^+2^ |  | 36.37 | 1.04 | 1550.0911^+2^ | 1550.0986^+2^ H6N5SAc3-PROC |
|  | | | | **28** | 36.70 | 0.37 | 1485.5856^+2^ | 1485.5773^+2^ H7N5S2-PROC *OR* H5N5S2F2-PROC |
| **29** | 36.82 | 1.41 | 1421.5702^+2^ |  | | | | 1421.5662^+2^ H8N5F2-PROC |
|  | | | | **29** | 37.00 | 0.30 | 1485.5488^+2^ | 1485.5773^+2^ H7N5SAc2-PROC *OR* H5N5SGc2F2-PROC |
| **30** | 37.29 | 4.8 | 1421.0616^+2^ |  | | | | 1421.056^+2^ H8N5SAc1-PROC |
|  | | | | **30** | 37.35 | 0.73 | 1493.5673^+2^ | 1493.5747^+2^ H7N5SAc1SGc1-PROC  *OR* H6N5SGc2F1-PROC |
|  |  |  |  |  |  |  | 1514.5854^+2^ | 1514.5982^+2^ H7N6SAc1F1-PROC |
|  |  |  |  | **31** | 37.65 | 1.92 | 1522.5850^+2^ | 1522.5957^+2^ H7N6SGc1F1-PROC |
|  |  |  |  |  |  |  | 1550.0969^+2^ | 1550.0986^+2^ H6N5SAc3-PROC |
|  |  |  |  | **32** | 38.14 | 2.04 | 1493.5712^+2^ | 1493.5747^+2^ H7N5SAc1SGc1-PROC  *OR* H6N5SGc2F1-PROC |
| **31** | 38.28 | 15.94 | 1429.5656^+2^ |  | | | | 1429.5636^+2^ H9N5F1-PROC |
|  | | | | **33** | 38.51 | 4.09 | 1522.5885^+2^ | 1522.5957^+2^ H7N6SGc1F1-PROC |
|  |  |  |  |  |  |  | 1034.7550^+3^ | 1034.7468^+3^ H8N7F1-PROC |
| **32** | 38.62 | 2.99 | 1429.5642^+2^ |  | | | | 1429.5636^+2^ H9N5F1-PROC |
|  |  |  | 1502.0807^+2^ |  |  |  |  | 1502.0824^+2^ H8N5SGc1F1-PROC |
|  |  |  | 1510.5930^+2^ |  |  |  |  | 1510.5901^+2^ H10N5F1 |
| **33** | 39.13 | 0.98 | 1493.5732^+2^ | **34** | 39.12 | 3.01 | 1493.5715^+2^ | 1493.5747^+2^ H6N5SGc2F1-PROC |
| **34** | 39.48 | 6.98 | 1502.0792^+2^ |  | | | | 1502.0824^+2^ H8N5SGc1F1-PROC |
| **35** | 40.07 | 1.77 | 1574.5934^+2^ |  |  |  |  | 1574.6012^+2^ H7N5SGc2F1-PROC |
| **36** | 40.37 | 0.98 | 1574.6067^+2^ |  |  |  |  | 1574.6012^+2^ H7N5SGc2F1-PROC |
|  |  |  | 1301.1949^+3^ |  |  |  |  | 1301.1633^+3^ H7N6SAc4F1-PROC |
| **37** | 40.87 | 0.35 | Not detected |  |  |  |  |  |
| **38** | 41.18 | 1.83 | 1574.5933^+2^ |  | | | | 1574.6012^+2^ H7N5SGc2F1-PROC |
|  | | | | **35** | 41.15 | 0.96 | 1676.1304^+2^ | 1676.1408^+2^ H8N6SAc1SGc1-PROC  *OR* H7N6SGc2F1-PROC |
| **39** | 41.36 | 2.73 | 1574.5797^+2^ |  | | | | 1574.6012^+2^ H7N5SGc2F1-PROC |
|  |  |  | 1612.1221^+2^ |  |  |  |  | 1612.1297^+2^ H10N6F1-PROC |
|  |  |  | 1623.1171^+2^ |  |  |  |  | 1623.1275^+2^ H6N5SAc3F1-PROC |
|  | | | | **36** | 41.47 | 0.37 | 1137.1028^+3^ | 1137.1103^+3^ H9N7SAc1-PROC *OR* H8N7SGc1F1-PROC |
| **40** | 41.93 | 1.09 | 1647.1157^+2^ | **37** | 41.91 | 0.65 | 1647.1333^+2^ | 1647.1199^+2^ H6N5SGc3F1-PROC |
|  |  |  | 1685.1830^+2^ |  | | | | 1685.1587^+2^ H10N6F2-PROC |
| **41** | 42.37 | 1.09 | 1684.6505^+2^ |  |  |  |  | 1684.6485^+2^ H10N6SAc1-PROC *OR*  H9N6SGcF1-PROC |
| **42** | 42.73 | 0.41 | 1647.6108^+2^ |  |  |  |  | 1647.6301^+2^ H7N5SGc2F2-PROC |
| **43** | 43.22 | 5.4 | 1693.1334^+2^ |  |  |  |  | 1693.1562^+2^ H11N6F1-PROC |
| **44** | 44.18 | 1.84 | 1765.6515^+2^ |  |  |  |  | 1765.6749^+2^ H11N6SAc1-PROC |
| **45** | 44.48 | 0.41 | 1838.1693^+2^ |  |  |  |  | 1838.1937^+2^ H10N6SAc1SGc1-PROC *OR* H9N6SGc2F1-PROC |
| **46** | 45.76 | 0.96 | *Not detected* | | | | | |

**Supplementary Table 5.** Comparison of Research Grade MOv18 IgE Untreated, R1, and Research Grade MOv18 IgE Alpha Galactosidase Treated samples, using technical Replicate 1 for each. Shown are predicted monosaccharide compositions and suggested glycan structures on observed MS m/z masses. H = hexose; N = N-acetylhexosamine; F = fucose; SGc = sialic acid (Neu5Gc); Sac = sialic acid (Neu5Ac).

**SUPPLEMENTARY REFERENCES**

1 *EudraLex The Rules Governing Medicinal Products in the European Union Volume 4 EU Guidelines to Good Manufacturing Practice Medicinal Products for Human and Veterinary Use Annex 13 Investigational Medicinal Products*, <<https://health.ec.europa.eu/document/download/eb43a2ab-4691-4cab-938e-874f2307dca3_en?filename=2009_06_annex13.pdf>> (

2 6.0, E. P. *Monoclonal antibodies for human use*, <<http://www.uspbpep.com/ep60/monoclonal%20antibodies%20for%20human%20use%202031e.pdf>> (

3 *ICH Topic Q 6 BSpecifications: Test Procedures and Acceptance Criteria for Biotechnological/Biological Products*, <<https://www.ema.europa.eu/en/documents/scientific-guideline/ich-q-6-b-test-procedures-and-acceptance-criteria-biotechnologicalbiological-products-step-5_en.pdf>> (

4 *EMA/CHMP/BWP/532517/2008 Committee for medicinal products for human use (CHMP) Guideline on development, production, characterisation and specification for monoclonal antibodies and related products*, <<https://www.ema.europa.eu/en/documents/scientific-guideline/guideline-development-production-characterisation-and-specification-monoclonal-antibodies-and-related-products-revision-1_en.pdf>> (

5 EMEA/CHMP/SWP/28367/07 Rev. 1 Committee for Medicinal Products for Human Use (CHMP) Guideline on strategies to identify and mitigate risks for first-in-human and early clinical trials with investigational medicinal products.

6 6.0, E. P. *Products of Recombinant DNA technology*, <<http://uspbpep.com/ep60/recombinant%20dna%20technology%20products%20of%200784e.pdf>> (

7 WHO. *WHO EXPERT COMMITTEE ON BIOLOGICAL STANDARDIZATION*, <<https://iris.who.int/bitstream/handle/10665/42013/WHO_TRS_878.pdf?sequence=1>> (
